# Inhibition of aortic CX3CR1^+^ macrophages mitigates thoracic aortic aneurysm progression in Marfan syndrome in mice

**DOI:** 10.1172/JCI178198

**Published:** 2025-01-16

**Authors:** Jiaqi Huang, Hao Liu, Zhujiang Liu, Zhenting Wang, Hanshi Xu, Zhuofan Li, Shan Huang, Xueyuan Yang, Yicong Shen, Fang Yu, Yulin Li, Junming Zhu, Wei Li, Li Wang, Wei Kong, Yi Fu

**Affiliations:** 1 Department of Physiology and Pathophysiology, School of Basic Medical Sciences, Peking University; State Key Laboratory of Vascular Homeostasis and Remodeling, Peking University, Beijing, China.; 2 Department of Cardiovascular Surgery, Beijing Anzhen Hospital of Capital Medical University, Beijing Institute of Heart Lung and Blood Vessel Diseases, Beijing Engineering Research Center of Vascular Prostheses, Beijing, China.; 3 State Key Laboratory of Cardiovascular Disease, Fuwai Hospital, National Center for Cardiovascular Diseases, Chinese Academy of Medical Sciences and Peking Union Medical College, Beijing, China.; 4 Beijing Anzhen Hospital, Capital Medical University, The Key Laboratory of Remodeling-Related Cardiovascular Diseases, Ministry of Education, Beijing Institute of Heart, Lung and Blood Vessel Diseases, Beijing, China.; 5 Department of Vascular Surgery, Peking University People’s Hospital, Beijing, China.

**Keywords:** Inflammation, Vascular biology, Cardiovascular disease, Macrophages

## Abstract

The pathogenesis of thoracic aortic aneurysm (TAA) in Marfan syndrome (MFS) is generally attributed to vascular smooth muscle cell (VSMC) pathologies. However, the role of immune cell–mediated inflammation remains elusive. Single-cell RNA sequencing identified a subset of CX3CR1^+^ macrophages mainly located in the intima in the aortic roots and ascending aortas of *Fbn1^C1041G/+^* mice, further validated in MFS patients. Specific elimination of CX3CR1^+^ cells by diphtheria toxin in *Cx3cr1*-CreER^T2^iDTR^F/+^*Fbn1^C1041G/+^* mice efficiently ameliorated TAA progression. Administering the monoclonal antibodies to respectively neutralize TNF-α and IGF1 produced by CX3CR1^+^ cells from MFS patients greatly suppressed the cocultured MFS patient–specific induced pluripotent stem cell–derived VSMC inflammation. BM transplantation and parabiosis revealed that CX3CR1^+^ macrophages are mainly originated from BM-derived monocytes. Targeting TNF-α and IGF1 in CX3CR1^+^ macrophages via shRNA lentivirus transduction in BM cells efficiently suppressed TAA development in BM-transplanted *Fbn1^C1041G/+^* mice. Application of the CCR2 antagonist RS504393 to inhibit monocyte infiltration markedly reduced the accumulation of CX3CR1^+^ macrophages and subsequently alleviated TAA progression in *Fbn1^C1041G/+^* mice. In summary, CX3CR1^+^ macrophages mainly located in aortic intima mediate TAA formation by paracrinally causing VSMC inflammation, and targeting them offers a potential antiinflammatory therapeutic strategy for MFS-related TAA.

## Abstract 2

Aortic CX3CR1^+^ macrophages derived from circulating monocytes mediate thoracic aortic aneurysm formation in Marfan syndrome by paracrinally causing vascular smooth muscle cell inflammation.

## Graphical abstract

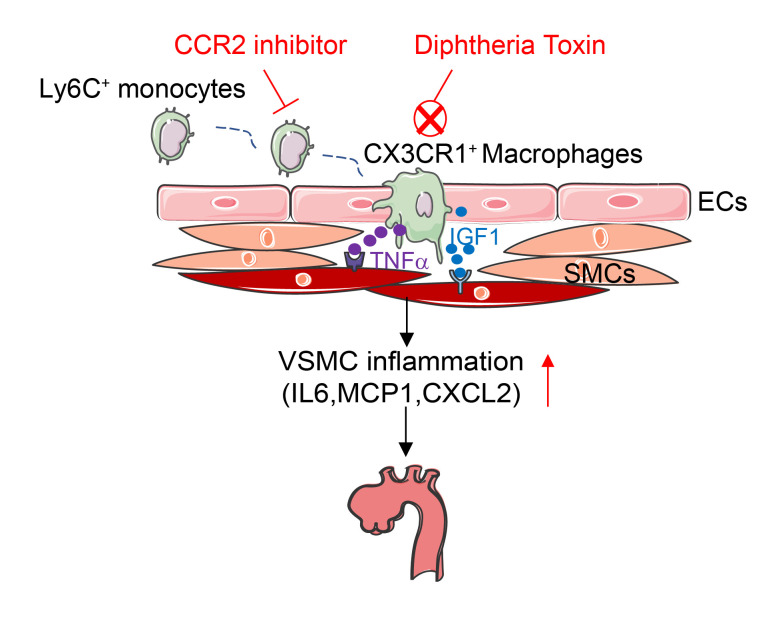

## Introduction

Marfan syndrome (MFS) is an autosomal dominant genetic disorder of the connective tissues caused by mutations in the fibrillin-1 (FBN1) gene (1, 2). Thoracic aortic aneurysm (TAA) is the most life-threatening manifestation of MFS due to the progression of acute aortic dissections and rupture causing lethal bleeding (3). To date, there are still a lack of effective therapies for TAAs in MFS, except for the administration of beta blockers with undefined efficacy and surgical repair with substantial morbidity/mortality risk (4). Although TGF-β and angiotensin II signaling pathways are well recognized to be involved in the pathogenesis of TAAs, the application of TGF-β neutralization and angiotensin II type I receptor (AT1R) antagonism in TAA treatment remains highly controversial, especially in some clinical trials (5–7). The unmet medical need is attributable to the limited knowledge on TAA etiology in MFS. Extensive mechanistic studies have mainly focused on alterations in vascular smooth muscle cells (VSMCs), such as VSMC phenotype modulation and impairment of mechanical properties (8, 9), whereas the roles of other cell types during the pathogenesis of TAAs in MFS are still far from understood. Thus, further exploration of the mechanism of TAA pathogenesis is essential to improve MFS intervention.

Immune cell–mediated vascular inflammation contributes to various vascular diseases, such as atherosclerosis, hypertension, and abdominal aortic aneurysm (AAA) (10–13), but is not well recognized as being involved in TAA etiology. Previous immunohistochemical staining revealed markedly increased numbers of T lymphocytes and macrophages in TAA tissues from MFS patients compared with aortic tissues from healthy controls (14), while a recent single-cell RNA-Seq (scRNA-Seq) study also suggested substantial infiltration of macrophages in TAA tissues from MFS mice and patients (15). However, whether inflammatory cells play a direct role in the pathogenesis of TAAs in MFS is still elusive.

In the current study, we identified a subset of macrophages highly expressing CX3CR1 that were substantially enriched in the aortic intima in MFS mice and patients and revealed that these CX3CR1^+^ macrophages were involved in the pathogenesis of TAAs in MFS mice by paracrinally causing VSMC inflammation. In addition, we clarified that intimal CX3CR1^+^ macrophages originated from circulating monocytes and that the inhibition of monocyte infiltration by a CCR2 inhibitor markedly reduced aortic CX3CR1^+^ macrophage numbers and subsequently mitigated TAA progression in MFS mice. Our study emphasized the pathogenic role of CX3CR1^+^ macrophages, which are mainly located in aortic intima, in TAA formation in MFS and indicated that antiinflammatory strategies targeting immune cells (e.g., intimal CX3CR1^+^ macrophages) would be promising in MFS therapy, especially for TAAs.

## Results

### CX3CR1^+^ macrophages were greatly enriched in the intima of thoracic aortas from MFS mice and patients.

The heterozygous *Fbn1*^C1041G/+^** mice were utilized as an MFS mouse model. In line with previous studies (16, 17), *Fbn1*^C1041G/+^** mice did not exhibit significant alterations in body weight and blood pressure (Supplemental Table 1; supplemental material available online with this article; https://doi.org/10.1172/JCI178198DS1), but spontaneously developed aortic root and ascending aortic aneurysms, as evidenced by transthoracic echocardiography on *Fbn1*^C1041G/+^** mice and littermate WT controls at the ages of 6, 10, and 20 weeks (Supplemental Figure 1A). Substantial dilations of aortic roots and ascending aortas were observed in *Fbn1*^C1041G/+^** mice at 10 weeks, followed by progressive aggravations at 20 weeks. Ex vivo aortic assessment further confirmed the aneurysmal dilations in 20-week-old *Fbn1*^C1041G/+^** mice (Supplemental Figure 1B), while elastica van Gieson (EVG) staining displayed obvious aortic wall pathologies, including elastin fragmentation and aortic wall thickness (Supplemental Figure 1C).

Accordingly, we performed scRNA-Seq on isolated aortic root and ascending aortic tissues from 20-week-old *Fbn1*^C1041G/+^** mice (*n* = 6 mice) and littermate WT controls (*n* = 6 mice). Following the cell-filtering process (Supplemental Figure 2), *Fbn1*^C1041G/+^** and WT samples (total 10,335 individual cells) were combined for general cell annotations. We clarified endothelial cells (ECs, marker genes: Pecam1, Vwf, Tek, Vcam1, Cdh5, Kdr), VSMCs (marker genes: Acta2, Myh11, Tagln, Cnn1, Mylk, Smtn), fibroblasts (marker genes: Pdgfra, Col1a2, Col3a1, Twist2, Col1a1, Pdgfrb), mesothelial cells (marker genes: Msln, Wt1, Bnc1), neuronal cells (marker genes: Plp1, Nrxn1, Nrxn3), and various leukocytes (marker genes: Igkc, Myl7, Itgb2) (Supplemental Figure 3A). Splitting the analysis by genotypes, we compared the cell populations between *Fbn1*^C1041G/+^** and WT samples and identified that leukocytes among all detected cell types were substantially increased in *Fbn1*^C1041G/+^** mice (Supplemental Figure 3B). Further flow cytometric analysis of single-cell samples digested from aortic roots and ascending aortas from 20-week-old *Fbn1*^C1041G/+^** and WT mice validated the increased infiltration of leukocytes (CD45^+^) in aneurysmal aortas (Supplemental Figure 3C).

**Figure 1:**
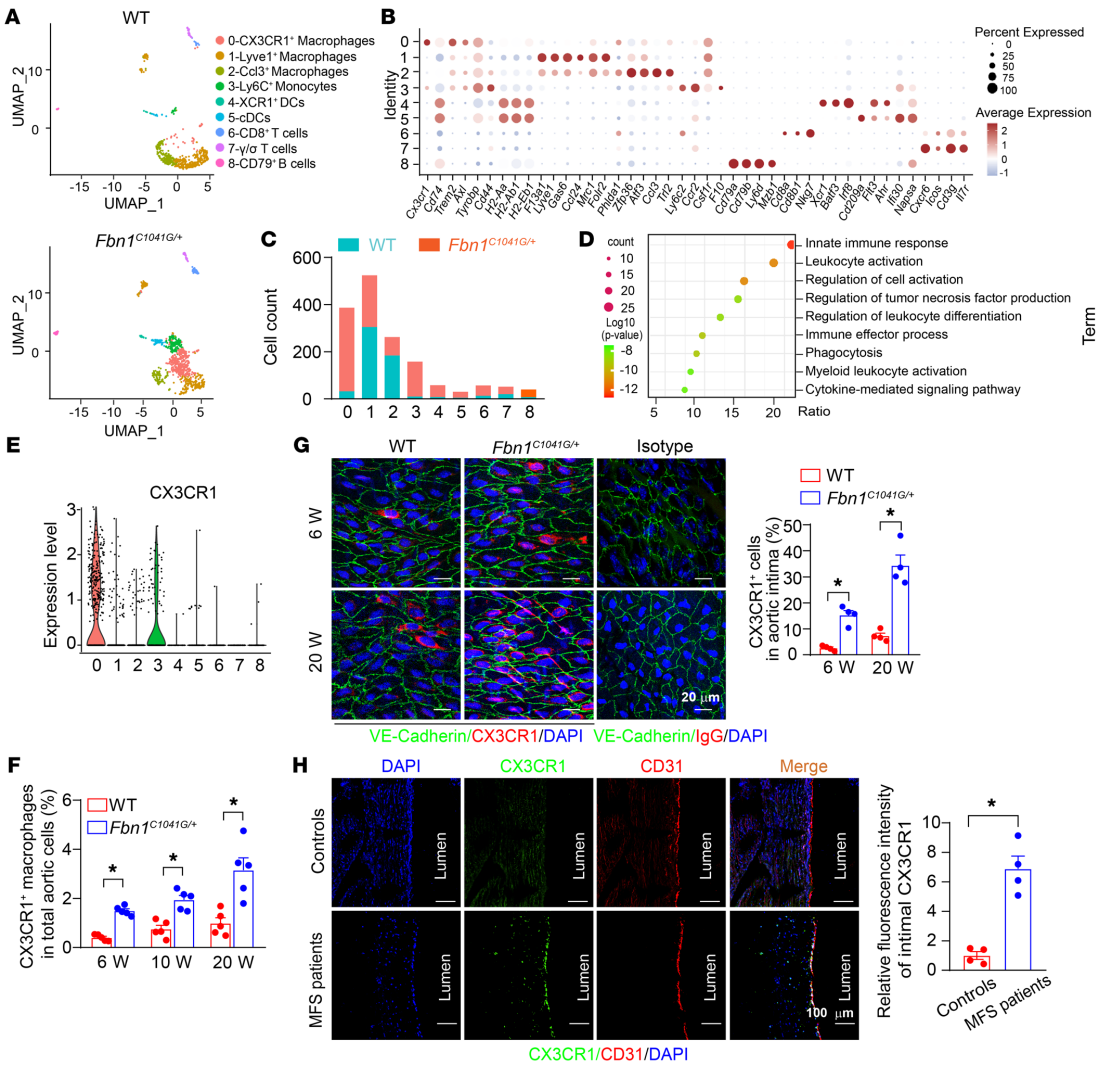
CX3CR1^+^ macrophages accumulated in the intima of thoracic aortas of MFS mice and patients. (**A**) UMAP visualization of leukocytes in aortic root and ascending aortas from 20-week-old WT and *Fbn1^C1041G/+^* male mice. (**B**) Selected marker gene expression in distinct leukocyte subtypes. (**C**) Cell counts in each leukocyte subtype. WT, *n* = 6,508 cells versus *Fbn1^C1041G/+^*, *n* = 3,827 cells. χ^2^ test to compare the cell quantitative changes. (**D**) GO analysis of genes enriched in CX3CR1^+^ macrophages (cluster 0). (**E**) The expression of CX3CR1 in distinct leukocyte subtypes. (**F**) Flow cytometry analysis of CX3CR1^+^ macrophages in aortic root and ascending aortas from WT and *Fbn1^C1041G/+^* mice at different ages. *n* = 5. *P < 0.05 by 2-way ANOVA followed by Tukey’s test for post hoc comparison. (**G**) En face immunofluorescence staining of VE-cadherin (green) and CX3CR1 (red) in the intima of ascending aortas from 6- or 20-week-old WT and *Fbn1^C1041G/+^* mice. The nuclei were stained blue with DAPI. Scale bars: 20 μm. Data were quantified as the percentages of CX3CR1^+^ cells (red cells) in total intima cells (the number of nuclei) averaged from 4 randomly selected areas of ascending aortas for each mouse. *n* = 4 mice. *P < 0.05 by 2-way ANOVA followed by Tukey’s test for post hoc comparison. (**H**) Immunofluorescence staining of CX3CR1 (green) and CD31 (red) in cross-sections of normal aortic tissues from control individuals (controls) and aneurysmal tissues from MFS patients. The nuclei were stained blue with DAPI. Scale bars:100 μm. Data were quantified as the relative fluorescence intensities of CX3CR1 staining in CD31 intimal areas averaged from 4 randomly selected areas for each patient. The average of intensities of CX3CR1 staining in normal controls was set as 1, while the intensities in MFS samples were presented as the relative values. *n* = 4. *P < 0.05 by unpaired Student’s *t* test.

Next, we performed a more thorough analysis to explore the subpopulations within the elevated leukocytes. The combined analysis of *Fbn1*^C1041G/+^** and WT samples identified 9 clusters of immune cells based on differentially expressed marker genes, including macrophages (clusters 0, 1 and 2), Ly6C^hi^ monocytes (cluster 3), dendritic cells (clusters 4 and 5), T cells (clusters 6 and 7), and B cells (cluster 8) (Figure 1, A and B). The marker genes for annotating each cluster are listed in Supplemental Data File 1. Comparing the cluster populations between the 2 genotypic samples, we found that CX3CR1^+^ macrophage numbers (cluster 0) were substantially elevated in the *Fbn1^C1041G/+^* aortas with the most abundant quantity (Figure 1C). Gene ontology (GO) analysis of marker genes expressed in CX3CR1^+^ macrophages showed enrichment of innate immune response, leukocyte activation, regulation of cell activation, regulation of tumor necrosis factor production, immune effector process, and phagocytosis, suggesting the proinflammatory role of these cells (Figure 1D). Furthermore, we deeply evaluated the specific expression of CX3CR1 among these 9 populations of leukocytes using scRNA-Seq data. As expected, cluster 0 (CX3CR1^+^ macrophages) exhibited the highest expression of CX3CR1 compared with other clusters (Figure 1E and Supplemental Figure 4A). Next, aortic single-cell flow cytometry validated that the number of CX3CR1^+^ macrophages (CD45^+^CD11b^+^F4/80^+^CX3CR1^+^) were elevated in *Fbn1^C1041G/+^* aneurysmal aortas as early as the age of 6 weeks and further increased with aging in MFS mice (Figure 1F). Furthermore, we compared the infiltration of CX3CR1^+^ macrophages in aneurysmal ascending aortas and roots with nonaneurysmal segments including descending aortas and abdominal aortas from 20-week-old *Fbn1^C1041G/+^* mice (Supplemental Figure 4B). Of note, CX3CR1^+^ macrophages were mainly accumulated in aortic roots and aneurysmal aortas in contrast to nonaneurysmal segments, suggesting a relatively specific association of CX3CR1^+^ macrophages with the pathogenesis of aortic aneurysm in MFS mice. Since *Fbn1^C1041G/+^* mice at the age of 6 weeks did not exhibit aortic dilation (Supplemental Figure 1A), the elevation of CX3CR1^+^ macrophages occurred prior to obvious aortic dilation, possibly exerting pathogenic effects on TAA formation.

To facilitate CX3CR1 evaluation, we crossbred *Cx3cr1*^GFP/+^*Fbn1^C1041G/+^* mice, in which CX3CR1^+^ cells were endogenously labeled with GFP (Supplemental Figure 5A). The development of TAAs was comparable between *Cx3cr1*^GFP/+^*Fbn1^C1041G/+^* mice and *Fbn1^C1041G/+^* mice at the age of 20 weeks (Supplemental Figure 5B). Cross-section immunofluorescence staining was performed to explore the location of CX3CR1^+^ macrophages in aortic walls from *Cx3cr1*^GFP/+^*Fbn1^C1041G/+^* mice at the age of 20 weeks. CX3CR1^+^ cells expressing GFP were mainly enriched in the aortic intima rather than the medial and adventitial layers in MFS mice (Supplemental Figure 5C). Considering the potential interruption of autofluorescence in elastic fibers on GFP signaling, we additionally performed intimal and adventitial en face immunofluorescence staining using anti-CX3CR1 antibody to validate the intimal location of CX3CR1^+^ macrophages. As a consequence, CX3CR1^+^ cells were markedly elevated in the aortic intima of *Fbn1^C1041G/+^* mice at the ages of 6 weeks and 20 weeks (Figure 1G). Of interest, substantial macrophages were observed in the aortic adventitia, but did not involve obvious CX3CR1 expression, supporting the increased CX3CR1^+^ cells in MFS mice mainly located in the aortic intima (Supplemental Figure 6A). Since previous studies suggested CX3CR1 mainly expresses in macrophages/monocytes and T cells among various leukocytes (18), we accordingly explored whether intimal CX3CR1^+^ leukocytes were macrophages (CD68) or T cells (CD3). As a result, more than 90% intimal CX3CR1^+^ cells expressed CD68, but did not exhibit substantial CD3 expression (Supplemental Figure 6B). Thus, most of the intimal CX3CR1^+^ cells were macrophages. To further clarify the topology of CX3CR1^+^ cells in relation to aortic intima, we first generated a 3D rendering of aortic intima using en face staining of intimal CX3CR1 and VE-cadherin. As a result, intimal CX3CR1^+^ cells were either above or interwoven in the endothelial layer (Supplemental Figure 6C). Meanwhile, flow cytometry data exhibited approximately 30% CX3CR1^+^ cells that were efficiently detected by intravascular injection of anti-CD45 antibodies in vivo, suggesting the luminal location of CX3CR1^+^ cells, whereas the remaining undetectable CX3CR1^+^ cells suggested their subintimal location (Supplemental Figure 6, D and E).

Furthermore, we collected aortic tissues from MFS patients and control individuals (Supplemental Table 2) and validated that the proportion of intimal CX3CR1^+^ macrophages were substantially increased in aneurysmal tissues compared with normal aortic tissue (Figure 1H and Supplemental Figure 7).

### Elimination of aortic CX3CR1^+^ cells ameliorated TAAs in Fbn1^C1041G/+^ mice.

**Figure 2:**
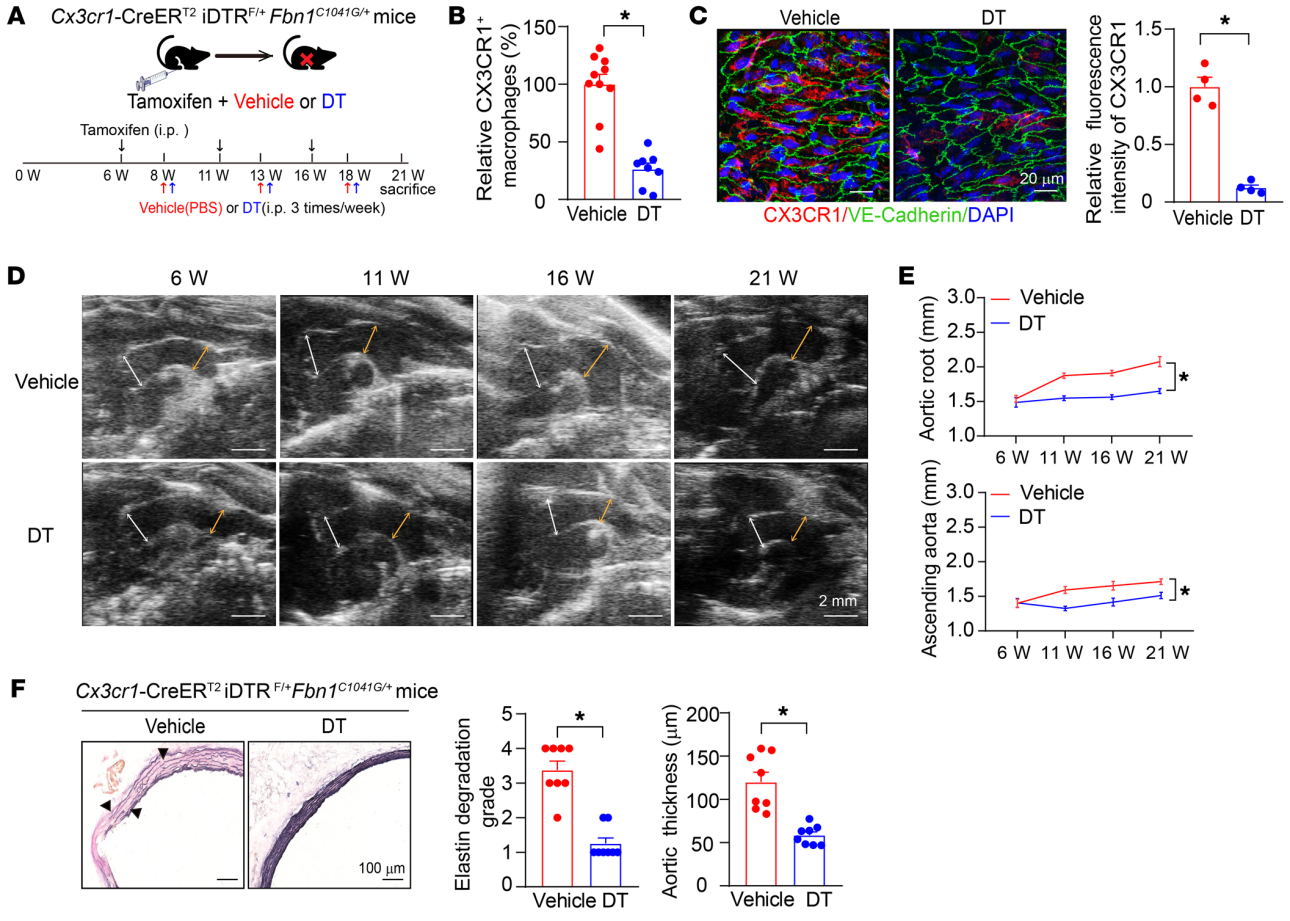
Depletion of aortic CX3CR1^+^ cells alleviated TAAs in *Fbn1^C1041G/+^* mice. (**A**) Experimental workflow of eliminating intimal CX3CR1^+^ macrophages to observe TAA development in *Cx3cr1*-CreER^T2^iDTR^F/+^*Fbn1^C1041G/+^* mice. (**B**) Flow cytometry analysis of CX3CR1^+^ macrophages in aortic root and ascending aortas from 21-week-old *Cx3cr1*-CreER^T2^iDTR^F/+^*Fbn1^C1041G/+^* mice with or without DT-mediated depletion. The average quantity of CX3CR1^+^ macrophages in the vehicle group was set as 100%, while that in the DT group was relative to that in the vehicle group. *n* = 9 for vehicle and *n* = 8 for DT. *P < 0.05 by unpaired Student’s *t* test. (**C**) En face immunofluorescence staining of intimal CX3CR1^+^ macrophages (red) and VE-cadherin (green) in ascending aortas from 21-week-old *Cx3cr1*-CreER^T2^iDTR^F/+^*Fbn1^C1041G/+^* mice with or without DT-mediated depletion. The nuclei were stained blue with DAPI. Scale bars: 20 μm. Data were quantified as the relative fluorescence intensities of CX3CR1 staining in intima averaged from 4 randomly selected areas for each mouse. The average of intensities of CX3CR1 staining in vehicle group were set as 1, while the intensities in DT group were presented as the relative values. *n* = 4 mice. *P < 0.05 by unpaired Student’s *t* test. (**D**) Representative transthoracic echocardiographic images of the aortic root and ascending aortas in *Cx3cr1*-CreER^T2^iDTR^F/+^*Fbn1^C1041G/+^* mice at different ages with or without DT-mediated depletion. Scale bars: 2 mm. White arrows depict the sinus of Valsalva measurements. Yellow arrows depict ascending aorta measurement. (**E**) Quantification of aortic root and ascending aorta diameters measured by transthoracic echocardiography. *n* = 10 for vehicle and *n* = 8 for DT. *P < 0.05 by repeated-measures ANOVA with the Greenhouse-Geisser adjustment followed by Bonferroni’s post hoc comparisons. (**F**) EVG staining of the aortic roots in 21-week-old *Cx3cr1*-CreER^T2^iDTR^F/+^*Fbn1^C1041G/+^* mice with or without DT-mediated depletion. *n* = 8 for each group. *P < 0.05 by Mann-Whitney *U* test for elastin degradation grade and unpaired Student’s *t* test for aortic thickness.

To explore the potential role of aortic CX3CR1^+^ macrophages in MFS-related TAA pathogenesis, we crossbred *Cx3cr1*-CreER^T2^iDTR^F/+^*Fbn1^C1041G/+^* mice (Supplemental Figure 8A). At the age of 6 weeks, these mice were intraperitoneally injected with tamoxifen (75 mg/kg/d, 5 days) to induce CX3CR1^+^ cells specifically expressing diphtheria toxin receptor (DTR), followed by intraperitoneal injection of diphtheria toxin (DT) (200 ng/20 g per time, 3 times in 1 week) to cause CX3CR1^+^ cell death and elimination. Since scRNA-Seq data displayed CX3CR1^+^ macrophages as well as Ly6C^hi^ monocytes substantially expressing CX3CR1 (Figure 1E and Supplemental Figure 4A), this depletion strategy mainly targeted these 2 populations of cells. A previous study indicated that the life term of intimal macrophages was approximately 1 month under pathological conditions (19); hence, we performed injections of tamoxifen and DT every 5 weeks to maintain the elimination of CX3CR1^+^ cells until the age of 21 weeks (Figure 2A). DT administration did not affect the body weight or blood pressure of mice (Supplemental Table 3). The elimination efficiency was evaluated by flow cytometry and immunofluorescence staining. Sequential injections of tamoxifen and DT efficiently reduced CX3CR1^+^ macrophages and monocytes, but without influencing aortic CX3CR1^–^ macrophage and lymphocyte infiltration, in 21-week-old *Cx3cr1*-CreER^T2^iDTR^F/+^*Fbn1^C1041G/+^* mice (Figure 2, B and C, and Supplemental Figure 8B), suggesting even additional depletion of some infiltrating monocytes seemed not to influence other CX3CR1^–^ cells, especially macrophages. In accordance, transthoracic echocardiography showed that DT-induced elimination of CX3CR1^+^ macrophages/monocytes greatly ameliorated both aortic root and ascending aortic aneurysm progression as well as related aortic wall pathologies in *Cx3cr1*-CreER^T2^iDTR^F/+^*Fbn1^C1041G/+^* mice (Figure 2, D–F). To exclude the potential off-target effect of DT on the development of aortic root and ascending aortic aneurysm, we administered DT in *Fbn1^C1041G/+^* mice (Supplemental Table 4). Consequently, DT did not directly affect aortic root and ascending aortic aneurysm progression in *Fbn1^C1041G/+^* mice (Supplemental Figure 8, C–E). Thus, these data collectively supported that aortic CX3CR1^+^ cells were involved in the pathogenesis of TAAs in MFS mice.

### CX3CR1^+^ macrophages caused VSMC inflammation by releasing TNF-α and IGF1.

**Figure 3:**
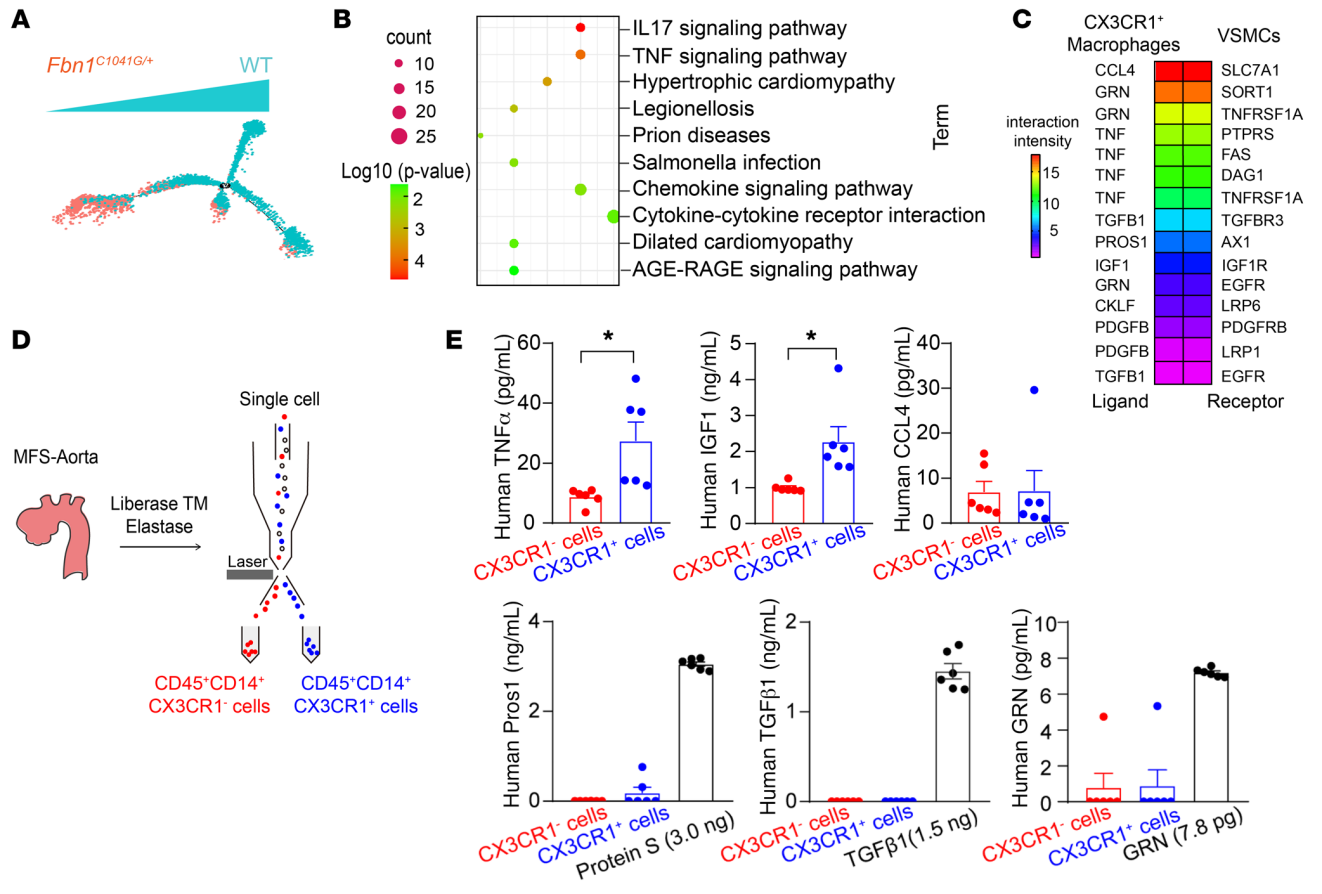
Ligand-receptor interactions between CX3CR1^+^ macrophages and VSMCs in MFS. (**A**) The pseudotime path of the VSMC transcriptome derived from the scRNA-Seq data on WT and *Fbn1^C1041G/+^* mice. (**B**) KEGG pathways enriched using the top 100 genes upregulated in *Fbn1^C1041G/+^* VSMCs compared with WT cells. (**C**) Bioinformatic analysis of ligand-receptor interactions between CX3CR1^+^ macrophages and VSMCs based on scRNA-Seq data. (**D**) The workflow of CX3CR1^+^ and CX3CR1^–^ macrophages isolated from MFS patients by FACS. (**E**) ELISA measurements of predicted ligand proteins secreted from CX3CR1^+^ or CX3CR1^–^ macrophages in conditioned media. *n* = 6. *P < 0.05 by unpaired Student’s *t* test.

Next, since the more significant elevation of CX3CR1^+^ macrophages (cluster 0) was observed in aortic roots and ascending aortas from MFS mice, compared with CX3CR1^+^ monocytes (cluster 3, Figure 1C), we preferentially deciphered how CX3CR1^+^ macrophages contributed to TAAs in MFS. As shown in Supplemental Figure 3, A and B, the clusters of ECs, VSMCs, and fibroblasts with abundant cell numbers did not exhibit obvious alterations in cell quantities or distinct subpopulations between *Fbn1*^C1041G/+^** and WT samples. We alternatively performed pseudotime trajectory analysis on ECs, VSMCs, and fibroblasts. ECs and fibroblasts did not show any difference between *Fbn1*^C1041G/+^** and WT cells (Supplemental Figure 9, A and B), whereas VSMCs in *Fbn1*^C1041G/+^** mice exhibited marked transcriptomic dynamics compared with WT cells (Figure 3A). Further Kyoto Encyclopedia of Genes and Genomes (KEGG) analysis of the top 100 upregulated genes in *Fbn1*^C1041G/+^** VSMCs showed enrichment of IL-17 signaling, TNF signaling, chemokine signaling, and cytokine-cytokine receptor pathways, suggesting inflammatory activation in VSMCs during TAA pathogenesis in MFS (Supplemental Data File 2 and Figure 3B). Therefore, we further verified the possibility that CX3CR1^+^ macrophages directly affected VSMC inflammation through cell-cell interaction analysis of scRNA-Seq data. As a result, 15 pairs of ligands produced by CX3CR1^+^ macrophages and receptors expressed on VSMCs were computationally predicted to elucidate the interaction between these 2 cell types (Figure 3C). Based on the interaction intensity, the top 6 ligands (CCL4, GRN, TNF-α, TGFβ1, PROS1, and IGF1) were selected for biological validation. We first isolated CX3CR1^+^ macrophages (CD45^+^CD14^+^CX3CR1^+^) and CX3CR1^–^ macrophages (CD45^+^CD14^+^CX3CR1^–^) as controls from thoracic aneurysmal aortas of MFS patients (Supplemental Table 5) and directly measured the predicted ligands in the conditioned media of these patient-derived cells (Figure 3D). Compared with control macrophages (CX3CR1^–^), CX3CR1^+^ macrophages produced more TNF-α and IGF1 in conditioned media, whereas other ligands were undetectable or were not altered (Figure 3E). In addition, our scRNA-Seq data also supported that TNF-α and IGF1 were most markedly upregulated in CX3CR1^+^ macrophages, rather than the other 2 clusters of macrophages from *Fbn1*^C1041G/+^** mice compared with WT controls (Supplemental Figure 9C).

Then, we also acquired patient-specific induced pluripotent stem cell–derived (iPSC-derived) VSMCs. MFS patient-specific iPSCs were generated from peripheral blood mononuclear cells through the ectopic expression of 4 transcription factors, OCT4, KLF4, SOX2, and cMyc (20) (Supplemental Figure 10A). Then, iPSCs from MFS patients were differentiated through lateral plate mesoderm (LM) progenitor stages followed by VSMC differentiation using a validated protocol (20, 21) (Supplemental Figure 10, B–D). The efficient differentiation of patient-specific iPSC-derived VSMCs was confirmed by the downregulation of iPSC and LM progenitor marker genes (iPSC: Supplemental Figure 10E and LM: Supplemental Figure 10F) as well as the upregulation of VSMC marker genes (Supplemental Figure 10G).

**Figure 4:**
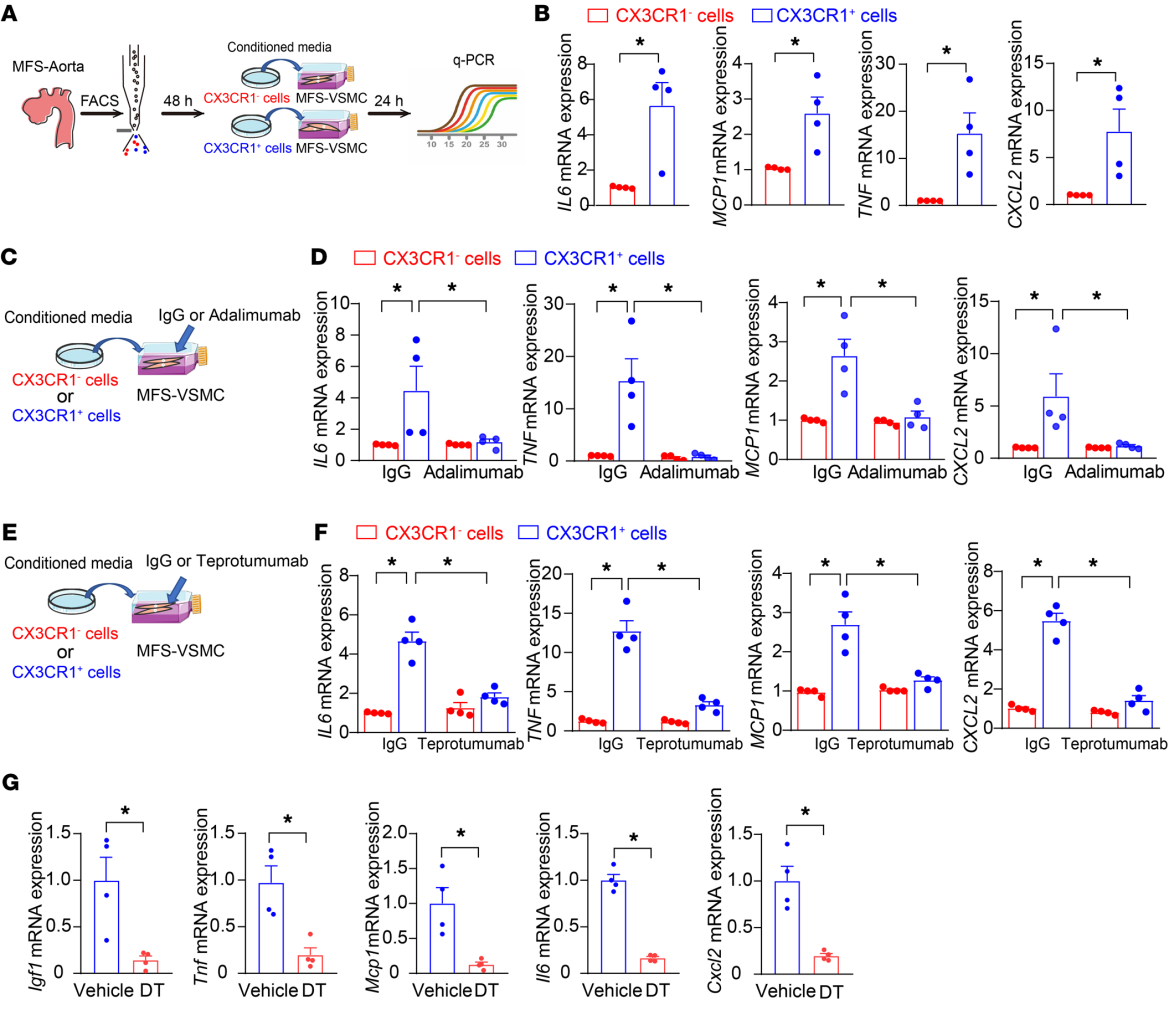
TNF-α– and IGF1-mediated intimal CX3CR1^+^ macrophage-caused VSMC inflammation. (**A**) The experimental workflow of the evaluation of gene expression in MFS patient-specific iPSC-derived VSMCs with 24-hour treatment of conditioned media produced from 48-hour cultures of MFS patient-derived CX3CR1^+^ and CX3CR1^–^ macrophages. (**B**) Real-time PCR of gene expression in MFS patient-specific iPSC-derived VSMCs with conditioned media treatment. *n* = 4. *P < 0.05 by unpaired Student’s *t* test. (**C** and **D**) Real-time PCR of gene expression in MFS patient-specific iPSC-derived VSMCs with conditional media treatment in the presence of adalimumab (10 μg/mL) or IgG. *n* = 4. *P < 0.05 by 2-way ANOVA followed by Tukey’s test for post hoc comparison. (**E** and **F**) Real-time PCR of gene expression in MFS patient-specific iPSC-derived VSMCs with conditioned media treatment in the presence of teprotumumab (200 μg/mL) or IgG. *n* = 4. *P < 0.05 by 2-way ANOVA followed by Tukey’s test for post hoc comparison. (**G**) Real-time PCR of gene expression in the aortic root and ascending aortas from 21-week-old *Cx3cr1*-CreER^T2^iDTR^F/+^*Fbn1^C1041G/+^* mice with or without DT-mediated depletion. *n* = 4. *P < 0.05 by unpaired Student’s *t* test.

To confirm the role of CX3CR1^+^ macrophages in VSMC inflammation, MFS patient–specific iPSC-derived VSMCs were treated with conditioned media from patient-derived macrophages, and then inflammatory genes enriched in *Fbn1*^C1041G/+^** VSMCs by pseudotime trajectory analysis in scRNA-Seq data were further evaluated in patient-derived VSMCs by quantitative PCR (qPCR) (Figure 4A). Consequently, conditioned media from CX3CR1^+^ macrophages, rather than CX3CR1^–^ cells, upregulated the expression of inflammatory genes (*IL6*, *MCP1*, *TNF*, and *CXCL2*) in VSMCs (Figure 4B). Moreover, the administration of adalimumab, a monoclonal antibody to neutralize TNF-α (22), or teprotumumab, a monoclonal antibody to block the IGF1 receptor (23) on VSMCs, inhibited VSMC inflammation induced by the conditioned media from CX3CR1^+^ macrophages (Figure 4, C–F). Next, we validated CX3CR1^+^ macrophage-related TNF-α– and IGF1-modulated VSMC inflammation in *Fbn1^C1041G/+^* mice with DT-caused depletion of aortic CX3CR1^+^ cells. As a result, elimination of CX3CR1^+^ cells thereby reducing TNF-α and IGF1 subsequently downregulated Mcp1, Il6, and Cxcl2 expression in aortic root and ascending aortas (Figure 4G). Of interest, we measured circulating TNF-α and IGF1 in *Fbn1^C1041G/+^* mice. Compared with WT controls, *Fbn1^C1041G/+^* mice did not exhibit the significant alteration of plasma TNF-α and IGF1 (Supplemental Figure 11), excluding the potential effects of TNF-α and IGF1 in blood circulation on aortic pathologies. These data suggested that CX3CR1^+^ macrophages perhaps enhanced VSMC inflammation in a paracrine manner in vivo.

### CX3CR1^+^ macrophages were derived from circulating monocytes during TAA pathogenesis in MFS mice.

Similar to other tissues, aortic macrophages can be either tissue resident or derived from circulating monocyte recruitment and differentiation (19, 24). To investigate the origins of aortic CX3CR1^+^ macrophages in MFS, we first crossed *Cx3cr1*^GFP/+^*Fbn1^C1041G/+^* mice (Supplemental Figure 5, A and B) and isolated bone marrow cells from *Cx3cr1*^GFP/+^*Fbn1^C1041G/+^* mice and injected them into lethally irradiated 8-week-old *Fbn1^C1041G/+^* mice (Supplemental Figure 12A). Twelve weeks after bone marrow transplantation, chimeric mice were validated for successful transplantation, as evidenced by the fact that approximately 80% of CX3CR1^+^ cells were GFP positive in peripheral blood and bone marrow (Supplemental Figure 12B). Moreover, substantial GFP-positive CX3CR1^+^ macrophages were identified in aneurysmal aortas from chimeric mice by flow cytometric analysis, suggesting that these cells were derived from bone marrow–derived circulating monocytes (Supplemental Figure 12B).

**Figure 5:**
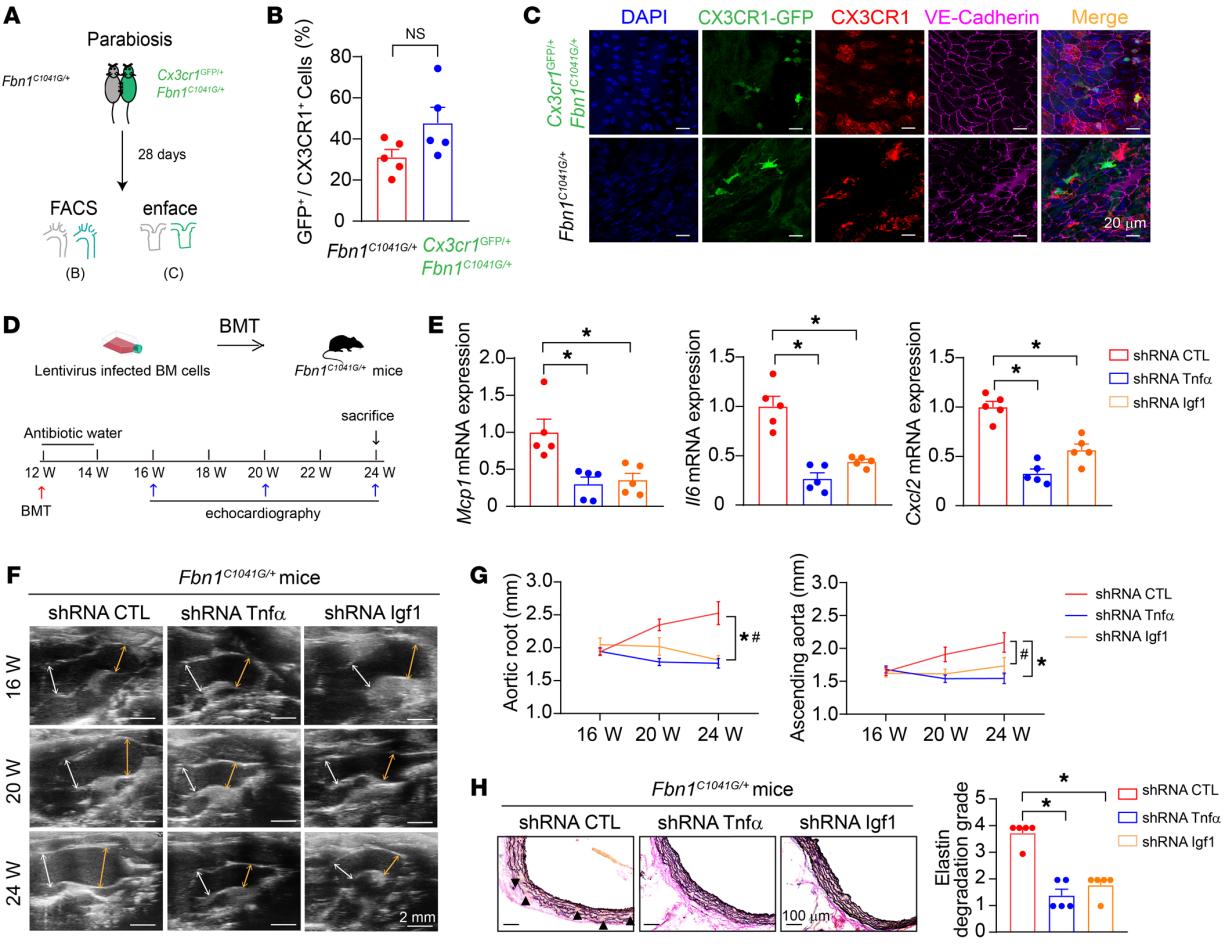
CX3CR1^+^ macrophages originated from bone marrow–derived circulating monocytes were involved in TAAs in *Fbn1^C1041G/+^* mice. (**A**) The experimental workflow of parabiosis of age-matched *Fbn1^C1041G/+^* mice and *Cx3cr1*^GFP/+^ *Fbn1^C1041G/+^* mice. (**B**) Flow cytometry analysis of GFP-expressing CX3CR1^+^ macrophages in aortic root and ascending aortas from *Fbn1^C1041G/+^* and *Cx3cr1*^GFP/+^ *Fbn1^C1041G/+^* parabionts. *n* = 5. Unpaired Student’s *t* test. (**C**) En face immunofluorescence staining of GFP-expressed intimal CX3CR1^+^ macrophages in ascending aortas from *Fbn1^C1041G/+^* and *Cx3cr1*^GFP/+^ *Fbn1^C1041G/+^* parabionts. Scale bars: 20 μm. (**D**) The experimental workflow of bone marrow transplantation (BMT) with *Fbn1^C1041G/+^* bone marrow cells infected with lentivirus encoding shRNA targeting TNF-α or IGF1 or control shRNA in 12-week-old *Fbn1^C1041G/+^* mice. (**E**) Real-time PCR of gene expression in aortic root and ascending aortas from *Fbn1^C1041G/+^* mice after BMT. *n* = 5. *P < 0.05 by 1-way ANOVA followed by Tukey’s test for post hoc comparison. (**F**) Representative transthoracic echocardiographic images of the aortic root and ascending aortas in *Fbn1^C1041G/+^* mice at different ages with control, Igf1, or Tnfα shRNA treatment. Scale bars: 2 mm. White arrows depict the sinus of Valsalva measurements. Yellow arrows depict ascending aorta measurement. (**G**) Quantification of aortic root and ascending aorta diameters measured by transthoracic echocardiography. *n* = 5. *^,#^*P* < 0.05 by repeated-measures ANOVA with the Greenhouse-Geisser adjustment followed by Bonferroni’s post hoc comparisons. (**H**) EVG staining of aortic roots in 24-week-old mice with control, Igf1, or Tnfα shRNA treatment. *n* = 5. *P < 0.05 by 1-way ANOVA followed by Tukey’s test for post hoc comparison.

Furthermore, we parabiosed 6-week-old *Cx3cr1*^GFP/+^*Fbn1^C1041G/+^* mice with age-matched *Fbn1^C1041G/+^* mice (Figure 5A). Four weeks later, the interparabiont blood circulation formed, and the paired mice shared leukocytes in circulation, as evidenced by the similar percentages of GFP-positive cells in peripheral blood (Supplemental Figure 12C). Flow cytometric analysis and en face immunofluorescence staining showed that the proportions of GFP-positive cells in intimal CX3CR1^+^ macrophages were comparable between the 2 parabionts (Figure 5, B and C: *Fbn1^C1041G/+^*, 31.13% ± 3.71% versus *Cx3cr1*^GFP/+^*Fbn1^C1041G/+^*, 47.79% ± 7.63%; *n* = 5, *P* = 0.34). These results indicated that intimal CX3CR1^+^ macrophages mainly originated from circulating monocyte recruitment and differentiation during TAA pathogenesis in MFS mice.

Since intimal CX3CR1^+^ macrophages were derived from bone marrow–derived circulating monocytes, we isolated bone marrow cells from *Fbn1^C1041G/+^* mice at the age of 6 weeks and infected the isolated cells with lentivirus encoding Tnfα or Igf1 shRNA, followed by transplantation of these infected bone marrow cells into 12-week-old aged *Fbn1^C1041G/+^* mice suffering from lethal irradiation, to achieve the efficient silencing of Tnfα or Igf1 in intimal CX3CR1^+^ macrophages at 4 weeks after bone marrow reconstitution, respectively (Figure 5D, Supplemental Figure 13, and Supplemental Table 6). In accordance, either knockdown of Tnfα or Igf1 in CX3CR1^+^ macrophages reduced vascular inflammation, as evidenced by the downregulation of Mcp1, Il6, and Cxcl2 (Figure 5E), and subsequently ameliorated both aortic root and ascending aortic aneurysm progression as well as related aortic wall pathologies (Figure 5, F–H). These results further strengthened the mechanism that CX3CR1^+^ macrophages aggravated MFS-related TAA progression through TNF-α– and IGF1-mediated vascular inflammation.

### Inhibition of circulating monocyte recruitment suppressed CX3CR1^+^ macrophage accumulation and TAA progression in MFS mice.

**Figure 6:**
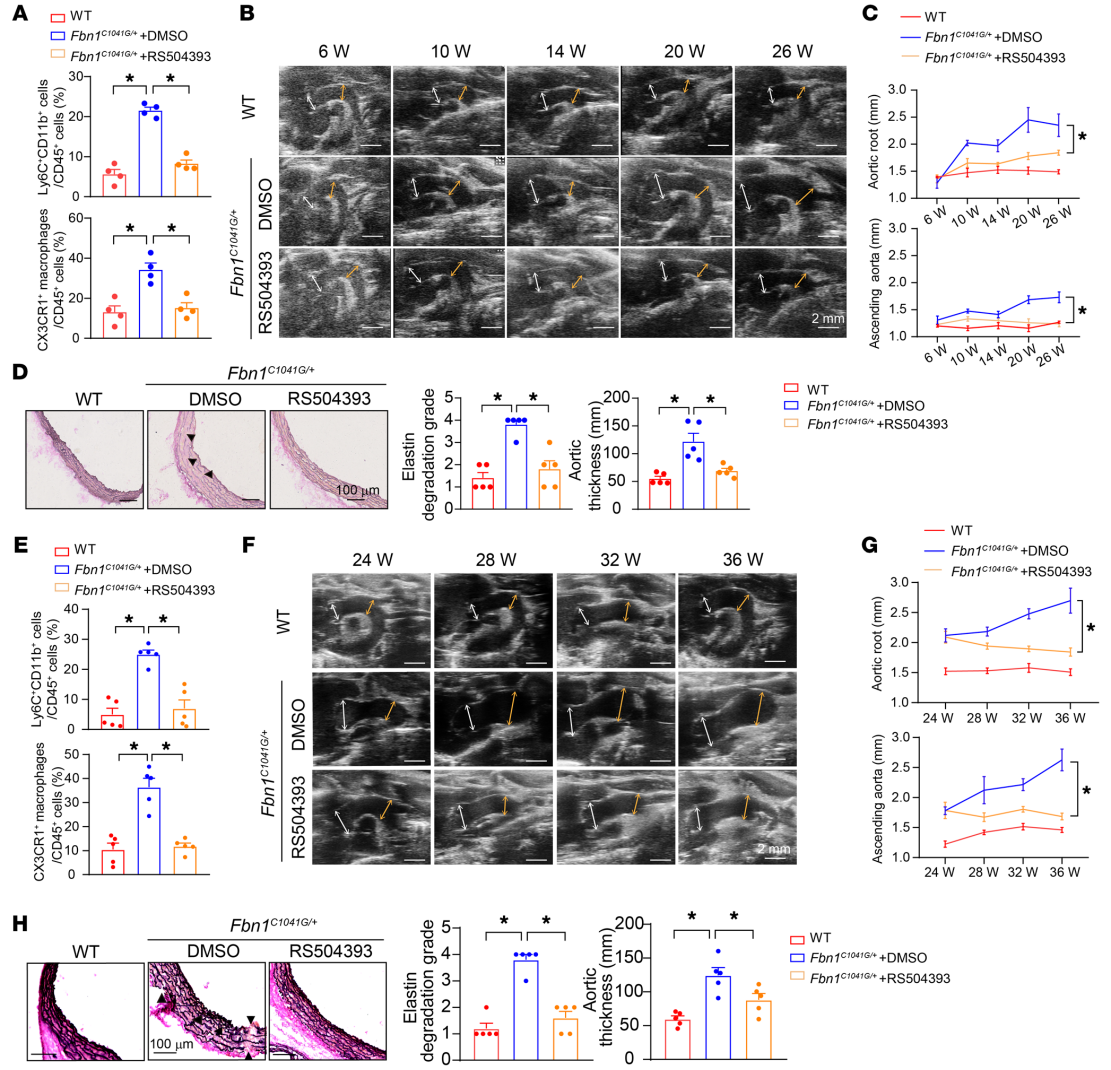
Suppression of CCR2 reduced CX3CR1^+^ macrophage accumulation and subsequently ameliorated TAAs in *Fbn1^C1041G/+^* mice. (**A**–**D**) Six-week-old *Fbn1^C1041G/+^* mice were intraperitoneally injected with RS504393 (4 mg/kg/d) until age 26 weeks. Age-matched WT mice were used as a normal control. (**A**) Flow cytometry analysis of Ly6C^+^ monocytes and CX3CR1^+^ macrophages in aortic root and ascending aortas. *n* = 4. *P < 0.05 by 1-way ANOVA followed by Tukey’s test for post hoc comparison. (**B**) Representative transthoracic echocardiographic images of the aortic root and ascending aortas. Scale bars: 2 mm. White arrows depict the sinus of Valsalva measurements. Yellow arrows depict ascending aorta measurement. (**C**) Quantification of transthoracic echocardiography. *n* = 4 for WT and *n* = 8 for *Fbn1^C1041G/+^* mice. *P < 0.05 by repeated-measures ANOVA with the Greenhouse-Geisser adjustment followed by Bonferroni’s post hoc comparisons. (**D**) EVG staining of the aortic roots. *n* = 5. *P < 0.05 by Kruskal-Wallis test followed by Dunn’s test for elastin degradation grade and 1-way ANOVA followed by Tukey’s test for post hoc comparison for aortic thickness. (**E**–**H**) Twenty-four-week-old *Fbn1^C1041G/+^* mice were intraperitoneally injected with RS504393 (4 mg/kg/d) until age 36 weeks. Age-matched WT mice were used as a normal control. (**E**) Flow cytometry analysis of Ly6C^+^ monocytes and CX3CR1^+^ macrophages in aortic root and ascending aortas. *n* = 5. *P < 0.05 by 1-way ANOVA followed by Tukey’s test for post hoc comparison. (**F**) Representative transthoracic echocardiographic images of the aortic root and ascending aortas. Scale bars: 2 mm. White arrows depict the sinus of Valsalva measurements. Yellow arrows depict ascending aorta measurement. (**G**) Quantification of transthoracic echocardiography. *n* = 5. *P < 0.05 by repeated-measures ANOVA with the Greenhouse-Geisser adjustment followed by Bonferroni’s post hoc comparisons. (**H**) EVG staining of aortic roots. *n* = 5. *P < 0.05 by Kruskal-Wallis test followed by Dunn’s test for elastin degradation grade and 1-way ANOVA followed by Tukey’s test for post hoc comparison for aortic thickness.

CCR2 is the major chemokine receptor mediating circulating monocyte recruitment during vascular inflammation (25). In addition, we also found the marked upregulation of MCP1 in aortic root and ascending aortas, where intimal CX3CR1^+^ macrophages mainly were located, rather than other segments of aortas in *Fbn1^C1041G/+^* mice prior to the age of 6 weeks, when we observed significant infiltration of intimal CX3CR1^+^ macrophages (Supplemental Figure 14). We thereby hypothesized that intimal CX3CR1^+^ macrophages were possibly derived from CCR2-mediated Ly6C^hi^ monocyte recruitment from circulation. Accordingly, we administered the CCR2 inhibitor RS504393 (4 mg/kg/d) to *Fbn1^C1041G/+^* mice to inhibit monocyte recruitment from the age of 6 weeks to 26 weeks. RS504393 did not affect body weights and blood pressures in *Fbn1^C1041G/+^* mice (Supplemental Table 7), but succeeded in inhibiting Ly6C^hi^ monocyte infiltration and subsequent CX3CR1^+^ macrophage accumulation without influencing other CX3CR1^–^ cells in thoracic aneurysmal aortas (Figure 6A and Supplemental Figure 15A), further supporting that CX3CR1^+^ macrophages originated from circulating Ly6C^hi^ monocyte recruitment. In accordance, both aortic root and ascending aortic aneurysms as well as related aortic wall pathologies were alleviated following RS504393 administration (Figure 6, B–D).

To further enhance the translational significance, we evaluated whether administration of CCR2 inhibitor RS504393 had therapeutic effects on established aneurysms. In accordance, RS504393 (4 mg/kg/d) was administered to *Fbn1^C1041G/+^* mice from the age of 24 weeks to 36 weeks (Supplemental Table 8). As a result, RS504393 also efficiently suppressed Ly6C^hi^ monocyte recruitment and subsequent CX3CR1^+^ macrophage accumulation in aneurysmal aortas (Figure 6E), thereby ameliorating the progression of aortic root and ascending aortic aneurysms in *Fbn1^C1041G/+^* mice (Figure 6, F–H, and Supplemental Figure 15B).

## Discussion

**Figure 7:**
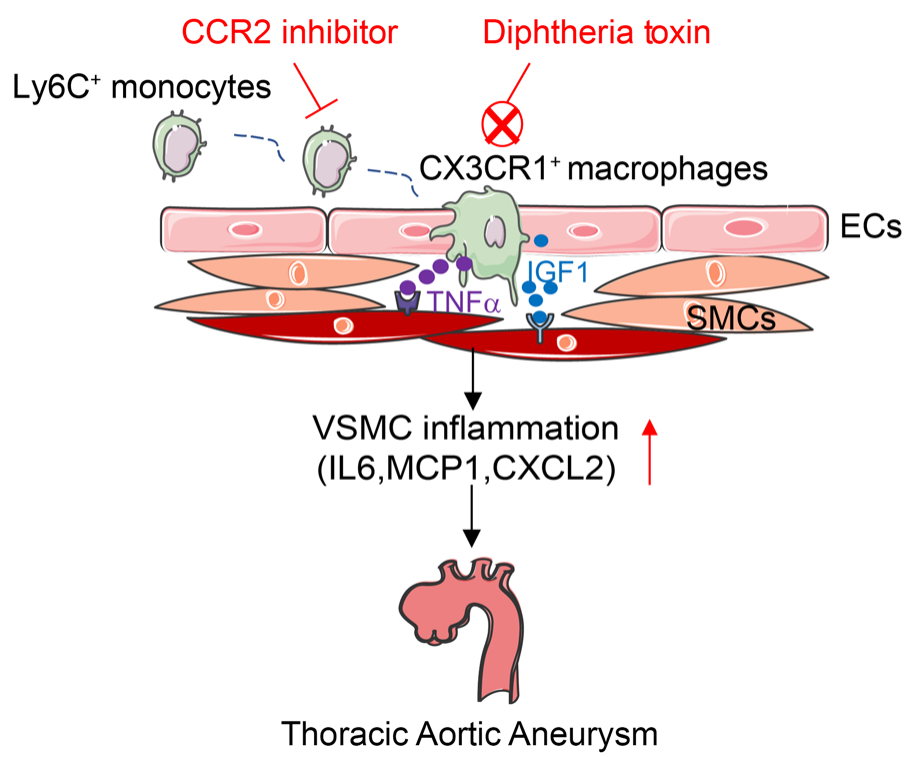
Schematic diagram of aortic CX3CR1^+^ macrophages that induce VSMC inflammation and promote TAA progression in MFS. CX3CR1^+^ macrophages mainly located in the aortic intima and originated from circulating Ly6C^+^ monocytes. Intimal CX3CR1^+^ macrophages upregulated inflammatory genes, including *IL6*, *MCP1*, and *CXCL2*, in VSMCs by producing and secreting TNF-α and IGF1, thereby aggravating TAA development in MFS. Either elimination of intimal CX3CR1^+^ macrophages or administration of a CCR2 inhibitor to suppress monocyte recruitment efficiently alleviated TAA progression in MFS.

In the current study, we revealed that intimal CX3CR1^+^ macrophages originating from circulating monocytes mediated the development of TAAs in MFS mice. Mechanistically, CX3CR1^+^ macrophages directly induced VSMC inflammation in a paracrine manner dependent on the proinflammatory cytokines TNF-α and IGF1. Both direct elimination of CX3CR1^+^ macrophages and administration of a CCR2 inhibitor to suppress monocyte recruitment efficiently alleviated TAA progression in MFS mice, suggesting that targeting intimal CX3CR1^+^ macrophage-mediated vascular inflammation may be a promising antiinflammatory strategy for treating TAAs in MFS (Figure 7).

The major finding of the current study is to reveal the critical role of immune cell–mediated inflammation in the pathogenesis of TAAs in MFS. Previous mechanistic exploration indicated that the etiology of MFS-related TAAs is mainly associated with VSMC phenotypic switching to further facilitate extracellular matrix degradation, impairment of vascular contractile ability, and vascular inflammation (15, 26, 27). Recently, dysregulation of nitric oxide (NO) signaling and disruption of tight junctions in ECs have been reported to be involved in TAA formation as well (28–31), suggesting the emerging role of ECs in TAA pathogenesis. Based on our scRNA-Seq data, we indeed found VSMC phenotypic switching to highly express proinflammatory cytokines, but did not observe significant alterations in EC populations or fibroblasts by cell trajectory analysis. Alternatively, we identified that a subtype of macrophages mainly located in the aortic intima and highly expressing CX3CR1 (intimal CX3CR1^+^ macrophages) was substantially enriched in the aortic roots and ascending aortas of *Fbn1^C1041G/+^* mice. In previous studies, whether immune cells contribute to TAA formation in MFS was not well recognized. Of note, a recent study clarified the pathogenic role of macrophages in a β-aminopropionitrile–induced (BAPN-induced) thoracic aortic aneurysm and dissection (TAAD) mouse model, as evidenced by the fact that suppression of macrophage accumulation in the aorta by using the CSF1R inhibitor Ki20227 decreased the incidence of TAAD and aortic rupture in mice (32). CSF1R inhibitors target the whole population of macrophages, but are not suitable to specifically intervene in the subset of CX3CR1^+^ macrophages. Alternatively, we incorporated DTR-mediated conditional and targeted cell ablation transgenic systems into *Fbn1^C1041G/+^* mice. Through the exogenous administration of DT, we efficiently eliminated CX3CR1^+^ macrophages and suppressed the development of TAAs. This finding provides direct evidence that CX3CR1^+^ macrophages drive the progression of TAAs in MFS. Of note, our macrophage-depletion strategy using DT also depleted CX3CR1^+^ macrophages in other tissues. Here, we identified that CX3CR1^+^ macrophages exerted their pathological action via producing TNF-α and IGF1. Of interest, we did not observe the significant elevation of TNF-α and IGF1 in circulating blood from *Fbn1^C1041G/+^* mice compared with WT controls, suggesting CX3CR1^+^ macrophages may exert pathogenic effects in a paracrine manner within the aortic wall, rather than the endocrine pathways and meanwhile excluding the possibility that other tissue CX3CR1^+^ macrophages took part in the pathogenesis of TAA in MFS in a TNF-α– and IGF1-dependent manner.

The crosstalk among vascular cells or between vascular cells and infiltrative immune cells plays a critical role in the development of vascular pathologies. EC-VSMC communication, that is, overactivation of soluble guanylate cyclase–cyclic GMP-dependent protein kinase G (sGC-PRKG) signaling in VSMCs by NO derived from ECs, has been reported to mediate aortopathy in MFS (33). Of note, the communication between macrophages and VSMCs has been confirmed to participate in atherosclerotic calcification and determine plaque stability. For example, macrophages deficient in cartilage oligomeric matrix protein produce procalcifying factors (e.g., Wnt10b) to induce VSMC osteogenic phenotype switching and atherosclerotic calcification (34). Moreover, M2 macrophages produce proresolving mediators (e.g., IL10 and TGF-β) and subsequently act on VSMCs to maintain atherosclerotic plaque stability, whereas M1 macrophages promote the development of rupture-prone plaques by releasing proinflammatory mediators (e.g., TNF-α and IL1β) and cause VSMC dedifferentiation (35). In the current study, we addressed the cell-cell communication between intimal CX3CR1^+^ macrophages and VSMCs that contributed to TAA formation in MFS. Based on the bioinformatic analysis of scRNA-Seq data from MFS mice, we directly explored cell-cell crosstalk in a coculture system using MFS patient–derived CX3CR1^+^ macrophages and MFS patient–specific iPSC-derived VSMCs, which may faithfully reflect the pathogenesis in MFS patients. We found that CX3CR1^+^ macrophages directly induced VSMC inflammation and aggravated TAA progression through the paracrine secretion of TNF-α and IGF1. The topology of CX3CR1^+^ macrophages in relation to the endothelial layer involved the luminal compartment, and intercellular/subintimal locations supported the possibility of the transendothelial movement of CX3CR1^+^ macrophages. Of note, following the transendothelial migration, the subintimal location might facilitate this proinflammatory interaction of CX3CR1^+^ macrophages with VSMCs. VSMC inflammation involving dedifferentiation and inflammatory gene upregulation is a common pathogenic event in aortic diseases, as conditional knockout of IL-6 or MCP1 in VSMCs alleviates the progression of both TAAs and AAAs in mice (27, 36, 37). Extensive studies have demonstrated that IGF1 obviously induces VSMC dedifferentiation through its receptor IGF1R signaling, while TNF-α is a well-known proinflammatory inducer that causes VSMC inflammation (38, 39). In addition, IGF1R signaling is also directly involved in inflammatory gene activation in CD34^+^ fibrocytes, as evidenced by IGF1-induced upregulation of IL-6 and IL-8 (23). Meanwhile, a recent study reported that administration of IGF1R antagonists protects against aortic aneurysm in rodent and porcine models, suggesting the pathogenic role of IGF1R signaling in aortopathy (40). Thus, TNF-α and IGF1, as the major mediators of intimal CX3CR1^+^ macrophage-induced VSMC inflammation, played the critical role in the pathogenesis of TAAs in MFS.

Aortic macrophages, as macrophages in other tissues, mainly have either tissue-resident or circulating monocyte-derived origins during the process of inflammation (41, 42). In our scRNA-Seq data, 3 subsets of macrophages (clusters 0–2) were annotated. Among them, cluster 1 macrophages highly expressed Lyve1, CCL24, and F13a1, similar to previously reported adventitia-resident macrophages in atherosclerotic mice (43). As in atherogenesis, this subset of macrophages was slightly enriched in the aortas of MFS mice but was not comparable with cluster 0 macrophages highly expressing CX3CR1, which was elevated most abundantly. Of interest, we found that CX3CR1^+^ macrophages were mainly located in the aortic intima. By using lineage-tracing technology in bone marrow transplantation and parabiosis models, we clarified that the accumulation of intimal CX3CR1^+^ macrophages depended on bone marrow–derived circulating monocytes. Meanwhile, we also observed a marked increase in the recruitment of Ly6C^hi^ monocytes with substantial CX3CR1 expression to aortas in MFS mice. Of interest, our scRNA-Seq suggested that Ly6C^hi^ monocytes (cluster 3) highly expressed CCR2 (Figure 1B), in line with previously published studies (44). Inhibition of CCR2 reducing intimal CX3CR1^+^ macrophages supported the possibility that intimal CX3CR1^+^ macrophages are mainly derived from circulating Ly6C^hi^ monocytes. Recently, a subpopulation of intima-resident macrophages highly expressing CX3CR1, similar to what we discovered in MFS mice and patients, has been identified in both physiological and atherosclerotic aortas from C57BL/6 mice (24, 41). These macrophages infiltrate the artery around birth and arise from circulating monocytes, subsequently adopting self-renewing capacity (24). Under physiological conditions, these intima-resident macrophages are essential for the maintenance of the nonthrombogenic intravascular state (41). Nevertheless, these cells are elevated following vascular pathologies, such as chemokine (e.g., MCP1) and cytokine (e.g., GM-CSF) secretion as well as matrix degradation (e.g., elastin-degrading peptides). Of note, the upregulation of MCP1 and GM-CSF as well as a GxxPG-containing fibrillin-1 fragment inducing macrophage chemotaxis has been found in the *Fbn1^mgR/mgR^* MFS mouse model (27, 45). Similarly, we also found that MCP1 was specifically upregulated in aortic root and ascending aorta segments in *Fbn1^C1041G^* mice before the age of 6 weeks, when we started to observe the elevation of intimal CX3CR1^+^ macrophage infiltration. Of note, it is well known that fibrillin-1 mutation enhances TGF-β bioavailability and downstream signaling activation (46–48). Previous studies have reported that TGF-β stimulation upregulated MCP-1 expression in both ECs and VSMCs through Smad3 signaling (49, 50). Thus, we could propose that fibrillin-1 mutation–facilitated TGF-β signaling promoted MCP-1 production in ECs and VSMCs and subsequently recruited circulating CCR2-expressed Ly6C^hi^ monocytes, which differentiated into macrophages, thereby favoring the accumulation of intimal CX3CR1^+^ macrophages during the pathogenesis of TAAs in MFS. Furthermore, our data also supported Ly6C^hi^ monocytes might mainly differentiate into CX3CR1^+^ macrophages, since DT-mediated depletion and CCR2 inhibition substantially suppressed the infiltration of CX3CR1^+^ cells involving CX3CR1^+^ macrophages and Ly6C^hi^ monocytes, rather than other CX3CR1^–^ cells. In accordance, we proposed Ly6C^hi^ monocytes might be involved in the pathogenesis of TAAs in MFS through differentiation into CX3CR1^+^ macrophages. Whether Ly6C^hi^ monocytes directly affect TAA progression will require further investigation.

The current treatment of TAAs in MFS is mainly restricted to beta blocker therapy and surgical repair. However, these therapies are associated with poor long-term outcomes and high mortality. Our current study revealed the driving role of intimal CX3CR1^+^ macrophage–mediated inflammation in TAAs, implying that antiinflammatory therapy might be a potential option to improve current treatment strategies for MFS. A CCR2 inhibitor succeeded in reducing intimal CX3CR1^+^ macrophage accumulation and alleviating the development of TAAs or even the established TAAs in MFS mice. Of interest, recent studies have reported that CCR2 deficiency or blockade additionally prevents myxomatous valve disease in MFS mice (51, 52), suggesting that antiinflammatory intervention on CCR2 may have multiple benefits for not only TAAs but also other manifestations of MFS. In addition, the therapeutic monoclonal antibodies adalimumab and teprotumumab, which have been utilized in the clinical treatment of autoimmune diseases and thyroid-associated ophthalmopathy (22, 23, 53), succeeded in blocking intimal CX3CR1^+^ macrophage–induced VSMC inflammation in the current study, suggesting that the application of adalimumab and teprotumumab targeting the proinflammatory cytokines produced by intimal CX3CR1^+^ macrophages could also be a potential MFS-related TAA therapy. Further clinical investigations on antiinflammatory therapy are needed to confirm the translation and application prospects.

Moreover, our study still has some additional limitations, which require further investigation to clarify in future. The first limitation is lacking of evidence in female *Fbn1^C1041G/+^* mice. Aiming to observe more obvious suppressive effects, we limited to including male *Fbn1^C1041G/+^* mice in the current study. Nevertheless, it is intriguing to explore whether the role of intimal CX3CR1^+^ macrophages exists in gender discrepancy. Second, *Fbn1^C1041G/+^* mice do not develop aortic dissection. Whether intimal CX3CR1^+^ macrophages affect aortic dissection requires further validation in another *Fbn1* mouse model, e.g., *Fbn1^mgR/mgR^* mice.

## Methods

### Sex as a biological variable.

Our study preferentially examined male *Fbn1^C1041G/+^* mice because male animals exhibited more severe aortopathies than the age-matched female ones (54–56), resulting in observation of more obvious inhibitory effects by intervention of intimal CX3CR1^+^ macrophages.

### Reagents.

Antibodies against mouse CD45 (Pacific blue labeled, 103126), mouse CD11b (APC labeled, 101212), mouse F4/80 (FITC labeled, 123107), mouse CX3CR1 (PE labeled, 149005; PerCP-Cyanine5.5 labeled, 149009), mouse CD3 (FITC labeled, 100203), mouse CD19 (AF700 labeled, 152413), and mouse CD144/VE-cadherin (A647 labeled,138006) were obtained from BioLegend. Antibodies against human CD45 (FITC labeled, 11-0459-41), human CD14 (PE Cyanine7 labeled, 25-0149-41), and human CX3CR1 (PE labeled, 12-6099-41) were obtained from Thermo Fisher. Antibodies against CX3CR1 (ab8020) and CD68 (ab955) were obtained from Abcam. Normal mouse IgG (68860L) and normal rabbit IgG (2729L) were obtained from Cell Signaling Technology. Alexa Fluor 488 goat anti-mouse IgG (A11001), Alexa Fluor 488 goat anti-rabbit IgG (A-11008), and Alexa Fluor 555 goat anti-rabbit IgG (A-21428) were obtained from Invitrogen. Collagenase type I (SCR103), collagenase type XI (C7657), hyaluronidase type I (H3506), and DNase I (10104159001) were obtained from Sigma-Aldrich. DT (23218) was purchased from Cayman Chemical. Tamoxifen (HY-13757A), adalimumab (HY-P9908), teprotumumab (HY-P99165), and the CCR2 inhibitor RS504393 (HY-15418) were purchased from MedChem Express. An elastin staining kit (BA4375B) was purchased from BASO Precision Optics Ltd. DAPI (C1002) was purchased from Beyotime Biotechnology. The OCT compound (4583) was obtained from SAKURA. Neomycin (AT0156) and polymyxin B sulfate (P105490) were purchased from Aladdin. The ELISA kits of human IGF1 (RK00160) and human TNF-α (RK00030) were purchased from ABclonal Technology Co. Ltd. The ELISA kits of human CCL4 (catalog KE00152) and human TGF-β1 (catalog KE00002) were purchased from Proteintech. The human granulin/GRN ELISA Kit (EK1184) was purchased from Multisciences (LIANKE) Biotech CO. Ltd. The human protein S ELISA Kit (CSB-E09903h) was purchased from CUSABIO. The ELISA kit of mouse TNF-α (QS42868) was purchased from Beijing Gersion Biotechnology Co. Ltd., and the ELISA kit of mouse IGF1 (YJ-E-30019M) was purchased from Yojobio Bio-Tech Co. Ltd.

### Mice.

A *Fbn1* mouse line (*Fbn1^C1041G/+^*) with the heterozygous C1041G mutation was obtained from George Tellides (Yale University School of Medicine, New Haven, Connecticut, USA) as previously described (17, 57) and was established by crossing the *Fbn1^C1041G/+^* male mice with WT C57BL/6J female mice. The mouse line *Cx3cr1*^GFP/+^*Fbn^C1041G/+^* was generated by crossing *Fbn1^C1041G/+^* male mice with *Cx3cr1*^GFP/+^ female mice (donated by Yu Nie). To generate the *Cx3cr1*-CreER^T2^iDTR^F/+^*Fbn1^C1041G/+^* mice, the *Cx3cr1*-CreER^T2^ mice (donated by Junwei Hao) were crossed with iDTR^F/F^ mice (obtained from The Jackson Laboratory, stock no.007900) to acquire *Cx3cr1*-CreER^T2^iDTR^F/+^ mice followed by further crossing with *Fbn1^C1041G/+^* mice. All mice were maintained on a C57BL/6 background. The detailed methods to measure aortic diameter and blood pressure as well as analyze aortic histological morphology can be found in Supplemental Methods.

### Single-cell sequencing analysis.

The detailed methods including aortic dissociation, scRNA-Seq, and related data analysis can be found in Supplementary Methods.

### Flow cytometry.

The dissociated aortic single-cell suspension, blood cells, and bone marrow cells were incubated with antibodies, including Pacific blue–labeled anti-CD45, APC-labeled anti-CD11b, FITC-labeled anti-F4/80, PerCP/Cyanine5.5-labeled anti-CX3CR1, and PE-labeled anti-Ly6C, as well as corresponding isotype IgGs for 30 minutes at 4°C. After washing away any unbound antibody using washing buffer (2% FBS, 0.05% EDTA in PBS), the cells were analyzed using a Beckman FACS Gallios system. CD45^+^CD11b^+^F4/80^+^CX3CR1^+^ cells were identified as CX3CR1^+^ macrophages, while CD45^+^CD11b^+^Ly6C^+^ cells were identified as Ly6C^+^ monocytes. Moreover, the detailed method to in vivo label the intimal CX3CR1^+^ macrophages for flow cytometry can be found in Supplemental Methods.

### Immunofluorescence staining.

Immunofluorescence staining was performed on aortic cross-sections or longitudinally opened thoracic aortas. Following antigen epitope retrieval with citrate solution, the samples were blocked with 3% bovine serum albumin for 1 hour at room temperature, incubated with rabbit anti-CX3CR1 antibodies (1:200 dilution, 2 μg/mL) and/or Alexa Fluor 647 anti-mouse VE-cadherin (1:200 dilution) overnight at 4°C, and then washed with PBS 3 times. Alexa Fluor 488 donkey anti-rabbit IgG (1:500 dilution, 2 μg/mL) secondary antibodies were incubated for 1 hour at 37°C and then washed with PBS 3 times, followed by nuclear staining with DAPI (1:2000 dilution, 5 μg/mL) for 10 minutes at room temperature. The longitudinally opened thoracic aortas were mounted on slides using antifade mounting solution, with the intima facing up. Immunofluorescence staining of arterial cross-sections or the endothelial layers of thoracic aortas was observed and acquired using the Leica Application Suite X system. The percentage of CX3CR1^+^ cells and fluorescence intensity of CX3CR1 staining were calculated and averaged from 6 sections of 5 randomly selected areas in each section.

### Cell.

For sorting human CX3CR1^+^ and CX3CR1^–^ macrophages, aortic root and descending aorta tissues were collected from MFS patients who underwent surgery. Single-cell suspensions from human MFS aortas were incubated with FITC-labeled anti-human CD45, PE-cyanine7–labeled anti-human CD14, and PE-labeled anti-human CX3CR1 as well as corresponding isotype IgGs for 30 minutes at 4°C. Following washing out unbound antibodies, cells were sorted by BD FACS Aria II SORP to collect CD45^+^CD14^+^CX3CR1^+^ cells as CX3CR1^+^ macrophages and CD45^+^CD14^+^CX3CR1^–^ cells as CX3CR1^–^ macrophages. These sorted cells were kept in RPMI 1640 medium with 10% FBS for 48 hours and conditioned media were collected for ELISA assay and further treatment of MFS patient-specific iPSC-derived VSMCs (with 50% conditioned media and 50% smooth muscle cell (SMC) cultured media for 24 hours). The details of MFS-iPSC derivation and VSMC differentiation can be found in Supplemental Methods.

### RT-qPCR analysis.

Total RNA was extracted using TRIzol Reagent (Vazyme). Equal amounts (1 μg) of RNA were reverse transcribed into cDNA by using NovoScript II Reverse Transcriptase (Novoprotein). SYBR Green 2× PCR mix (Novoprotein) was used according to the manufacturer’s instructions. Quantitative reverse-transcriptase PCR (RT-qPCR) analysis was performed by using an ABI QuantStudio 3 Real-Time PCR System. All amplification reactions were carried out with a program involving a step at 95°C for 1 minute followed by 40 cycles of 95°C for 15 seconds and 60°C for 1 minute. The data were analyzed using the ΔΔCT method with QuantStudio Design & Analysis Software and normalized with 18S as an internal control. The primer sequences for the target genes used for all RT-qPCR analyses are listed in Supplemental Table 9.

### Bone marrow transplantation and parabiosis.

Details can be found in Supplemental Methods.

### Statistics.

Data are presented as mean ± SEM. Statistical analysis was performed using GraphPad Prism, version 8.0, software (GraphPad Software). For statistical comparisons, we first evaluated whether the data were normally distributed using the Shapiro-Wilk normality test. For normally distributed data, unpaired, 2-tailed Student’s *t* test was utilized for 2-group comparisons, whereas ANOVA followed by post hoc comparisons was used in multiple group data analyses. If the data were not normally distributed, a nonparametric test was used. In all analyses, *P* < 0.05 was regarded as statistically significant. The detailed statistical analyses used for each experiment are presented in the corresponding figure legends.

### Study approval.

All MFS patients were diagnosed according to the revised Ghent nosology (58).The 4 non-MFS individuals served as controls, 3 of them had undergone dilated cardiomyopathy, and the other 1 had arrhythmia cardiomyopathy. All patients signed an informed consent form before surgery and sample collection. The study protocol was approved by Beijing Anzhen Hospital (KS2019016) in accordance with the principles of the Declaration of Helsinki. Detailed information about all the patients is listed in Supplemental Tables 2 and 5. All mouse experiments were approved (LA2021103) by the Institutional Animal Care and Use Committee of Peking University Health Science Center, and the care of the animals was in accordance with institutional guidelines.

### Data availability.

The high-throughput sequence data of scRNA-Seq has been uploaded to the NCBI’s Gene Expression Omnibus database (GEO GSE249352). Additional information can be obtained from the corresponding authors upon request. Values for all data points in graphs are reported in the Supporting Data Values file.

## Author contributions

JH performed the experiments and analyzed the results. HL generated and cultured iPSCs. Z Liu, ZW, and HX acquired and analyzed single-cell RNA sequencing. SH and YL performed and analyzed the echocardiography. XY and YS performed the immunofluorescence staining experiments. FY performed the flow cytometry analysis. Z Li, WL, and LW supported the transgenic mice experiments. JZ and HL provided human samples. LW, WK, and YF conceived the study, designed the experiments, and analyzed the results. JH and YF wrote the manuscript. HL, LW, WK, and YF edited the manuscript. All authors have discussed the results and had the opportunity to comment on the manuscript.

## Supplementary Material

Supplemental data

Supplemental data set 1

Supplemental data set 2

Supporting data values

## Version 1 01/16/2025

Electronic publication
