# Supplementary material for: Inhibition of aortic CX3CR1+ macrophages mitigates thoracic aortic aneurysm progression in Marfan syndrome in mice
Source: J Clin Invest. 2025 Jan 16;135(2):e178198. doi: 10.1172/JCI178198 (PMC11735105; doi:10.1172/JCI178198)
Supplement: Supplemental data [file jci-135-178198-s183.pdf]

Supplementary Materials

Inhibition of Aortic CX3CR1^{+} Macrophages Mitigates Thoracic Aortic Aneurysm Progression in Marfan Syndrome in Mice

Jiaqi Huang, Hao Liu, Zhujiang Liu, Zhenting Wang, Hanshi Xu, Zhuofan Li, Shan Huang, Xueyuan Yang, Yicong Shen, Fang Yu, Yulin Li, Junming Zhu, Wei Li, Li Wang, Wei Kong and Yi Fu

This PDF includes:

1. Supplementary Figure 1-15
2. Supplementary Table 1-10
3. Supplementary Methods
4. Supplementary References

Supplementary Figure 1

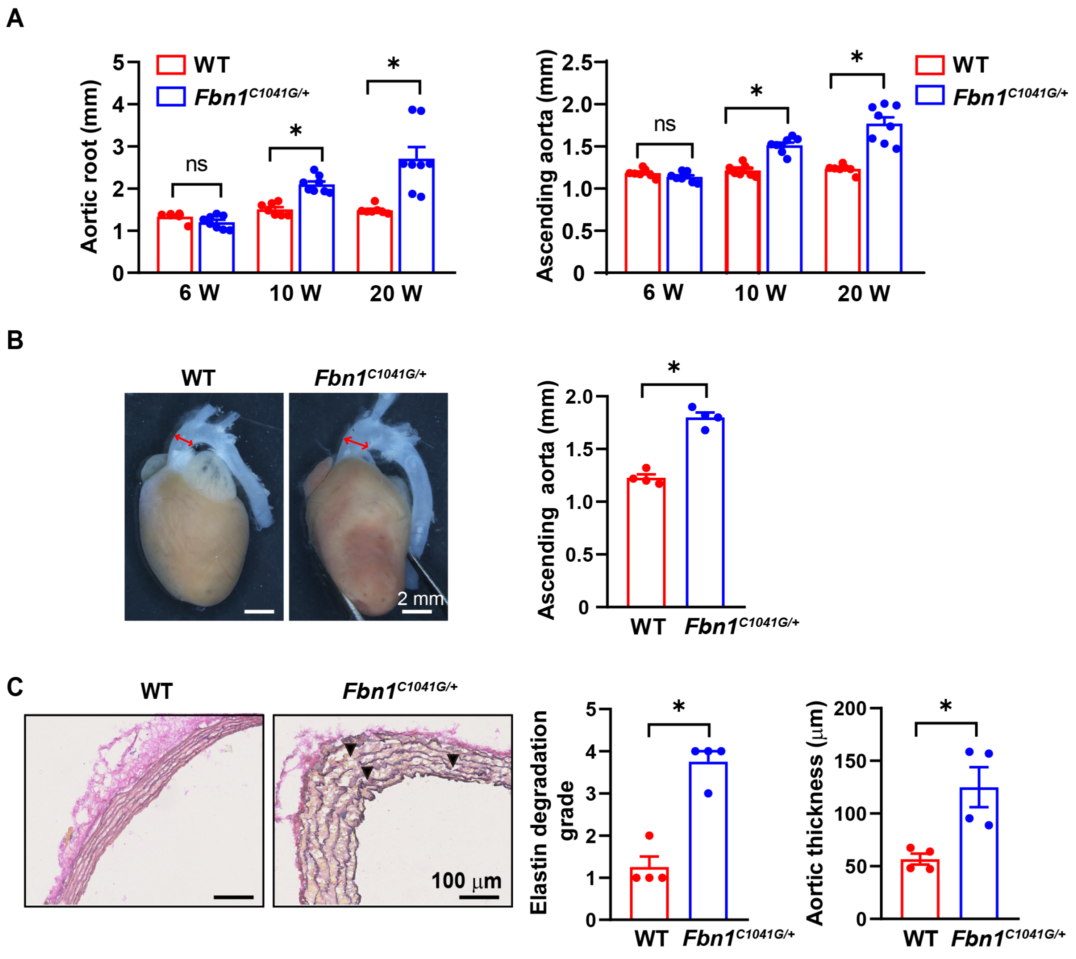

Supplementary Figure 1. Characteristics of thoracic aortas in WT and *Fbn1*$^{C1041G/+}$ mice.

A. Transthoracic echocardiographic measurement of aortic root and ascending aorta dimeters in WT and *Fbn1*$^{C1041G/+}$ mice at different ages. n=7 for WT and n=8 for *Fbn1*$^{C1041G/+}$ mice, *P<0.05 by two-way ANOVA followed by Tukey's test for post hoc comparison. **B.** *Ex vivo* visualization of ascending aorta expansions from 20-week-old WT and *Fbn1*$^{C1041G/+}$ mice. Scale bar=2 mm. n=4, *P<0.05 by unpaired Student's *t* test. **C.** EVG staining of the ascending aortas in 20-week-old WT and *Fbn1*$^{C1041G/+}$ mice. Scale bar=100 µm. n=4, *P <0.05 by Mann-Whitney test for elastin degradation grade and unpaired Student's *t* test for aortic thickness.

Supplementary Figure 2

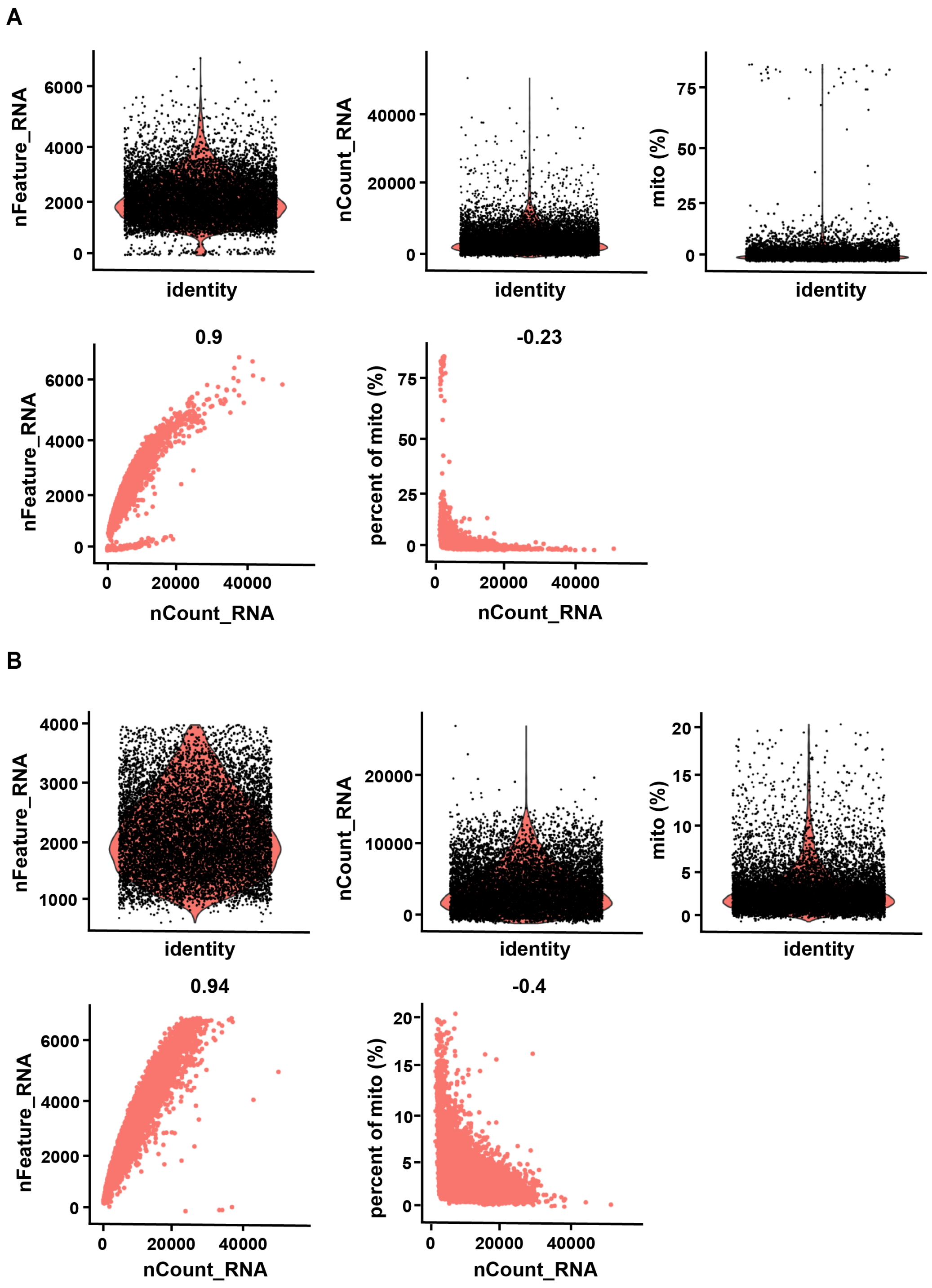

Supplementary Figure 2. The Quality Control (QC) metrics for scRNA-Seq data before (A) or after (B) cell filtrating process. The violin plots from the left to right respectively: the number of genes per cell (nFeature_RNA), the absolute number of observed transcripts per cell (nCount_RNA), and the percentage of transcripts arising from the mitochondria genome [mito (%)].

Supplementary Figure 3

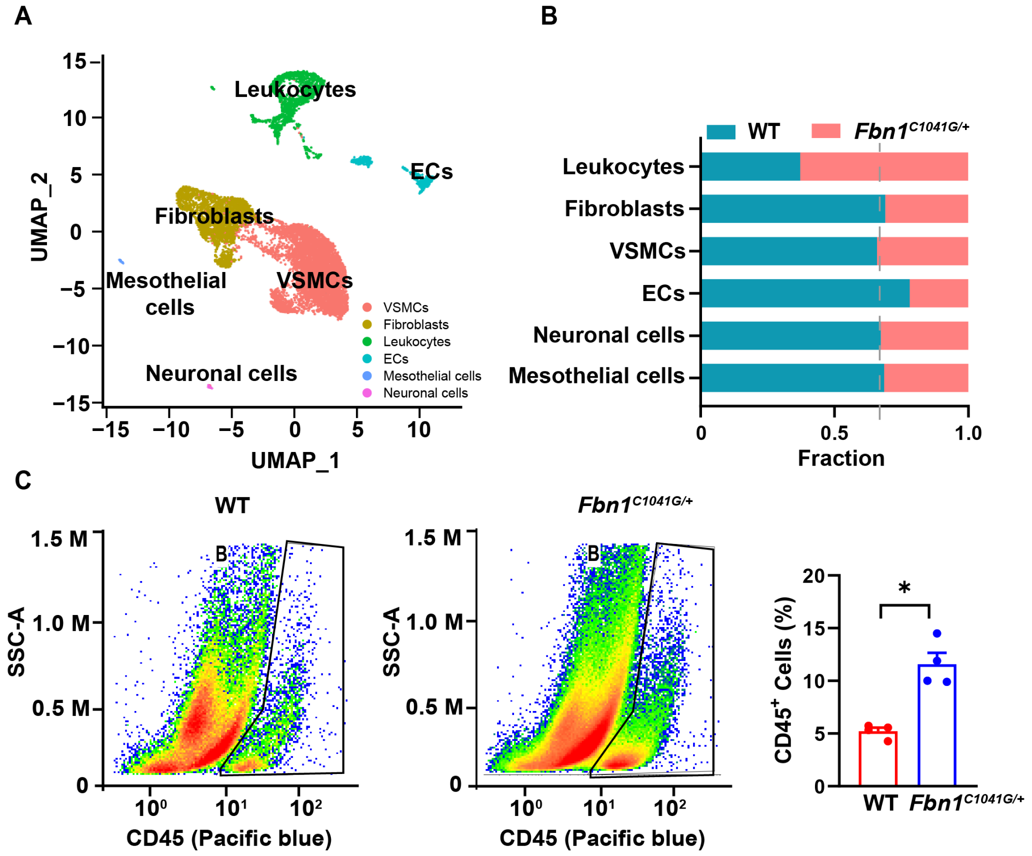

Supplementary Figure 3. Single-Cell RNA sequencing identified increased leukocytes in MFS mouse model. A. UMAP visualization of the major cell types in the aortic root and ascending aortas between WT and *Fbn1*$^{C1041G/+}$ male mice at 20 weeks of age. n=10,335 cells. **B**. Relative compositions calculated by the cell numbers in each cell type from 20-week-old WT and *Fbn1*$^{C1041G/+}$ male mice. The gray dashed line represents the expected proportion of cells from the WT group (total number of WT group cells divided by total number of cells from all mice, 6,508/10,335). **C.** The percentages of leukocytes ($CD45^+$) in total cells detected by flow cytometry in aortic root and ascending aortas from 20-week-old WT and *Fbn1*$^{C1041G/+}$ mice. n=4, *P<0.05 by unpaired Student's *t* test.

Supplementary Figure 4

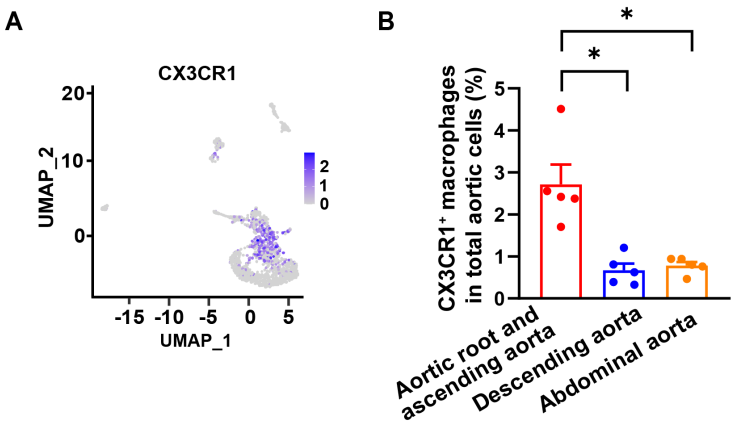

Supplementary Figure 4. A. UMAP visualization of CX3CR1 expression in different clusters of leukocytes. **B.** Flow cytometry analysis of CX3CR1$^+$ cells in different segments of aortas from 20-week-old *Fbn1*$^{C1041G/+}$ mice. n=5, *P<0.05 by unpaired Student's t test.

Supplementary Figure 5

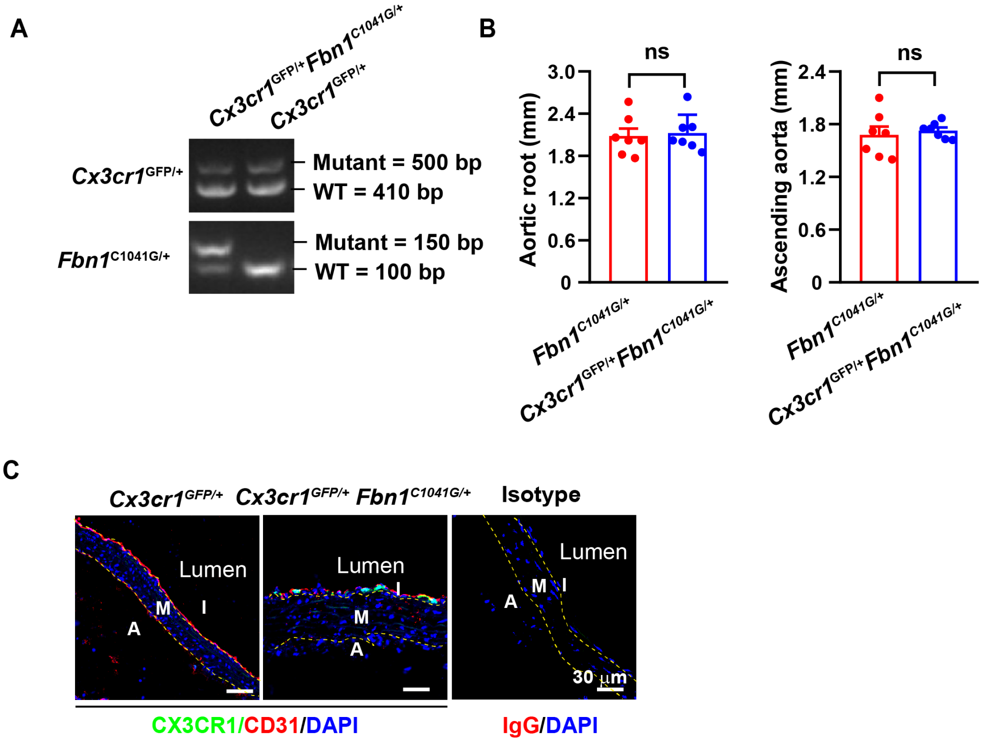

Supplementary Figure 5. A. Representative genotyping of *Cx3cr1*$^{GFP/+}$*Fbn1*$^{C1041G/+}$ and *Cx3cr1*$^{GFP/+}$ mice. **B**. Transthoracic echocardiographic measurement of aortic root and ascending aorta dimeters in *Cx3cr1*$^{GFP/+}$*Fbn1*$^{C1041G/+}$ and *Fbn1*$^{C1041G/+}$ mice at 20 weeks of age. n=7, unpaired Student's *t* test, ns, no significance. **C**. Immunofluorescence staining of CD31 (red) and CX3CR1 (green) in cross-sections of ascending aortas from 20-week-old *Cx3cr1*$^{GFP/+}$ and *Fbn1*$^{C1041G/+}$*Cx3cr1*$^{GFP/+}$ mice. The nuclei were stained blue with DAPI. Scale bar=30 µm. Rat IgG (red) as negative isotype controls. I, intima, M, media, A, adventitia.

Supplementary Figure 6

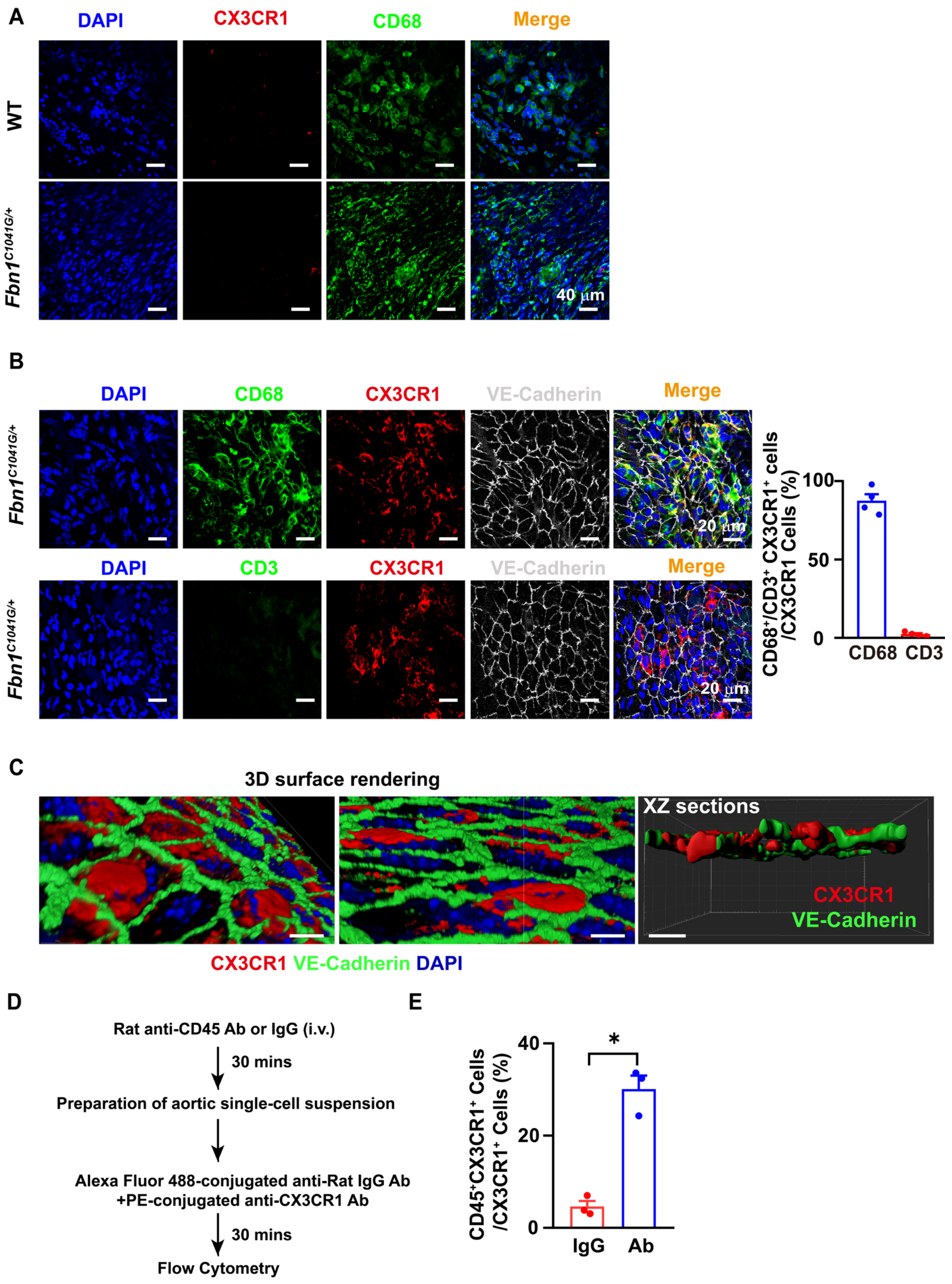

Supplementary Figure 6. ***En face*** **immunofluorescence staining of CX3CR1$^+$ cells in the intima and adventitia of ascending aortas from Fbn1$^{C1041G/+}$ mice. A.** *En face* immunofluorescence staining of CX3CR1 (red) and CD68 (green) in the adventitia of ascending

aortas from 20-week-old *Fbn1*$^{C1041G/+}$ mice. The nuclei were stained blue with DAPI. Scale bar=20 μm. n=4 mice. **B**. *En face* immunofluorescence staining of CX3CR1 (red) and CD68/CD3 (green) in the intima of ascending aortas from 20-week-old *Fbn1*$^{C1041G/+}$ mice. The nuclei were stained blue with DAPI. Scale bar=20 μm. Data were quantified as the percentages of CD68^{+}/CD3^{+} cells (green) in CX3CR1^{+} cells (red) averaged from 4 randomly selected areas of ascending aortas for each mouse. n=4 mice. **C.** *Fbn1*$^{C1041G/+}$ mice at the age of 20 weeks used for 3D surface rendering of ascending aortas to visualize the spatial location of intima CX3CR1^{+} cells (red) relative to the endothelial cells (green). The nuclei were stained blue with DAPI. Scar bars=5 μm. **D.** The experimental procedure of labelling CX3CR1^{+} macrophages in aortic root and ascending aortas in *Fbn1*$^{C1041G/+}$ mice at the age of 20 weeks. **E.** Flow cytometry analysis of the percentages of CD45^{+}CX3CR1^{+} cells locating luminal compartment in aortic CX3CR1^{+} cells from *Fbn1*$^{C1041G/+}$ mice. n=3, *P<0.05 by unpaired Student's t test.

Supplementary Figure 7

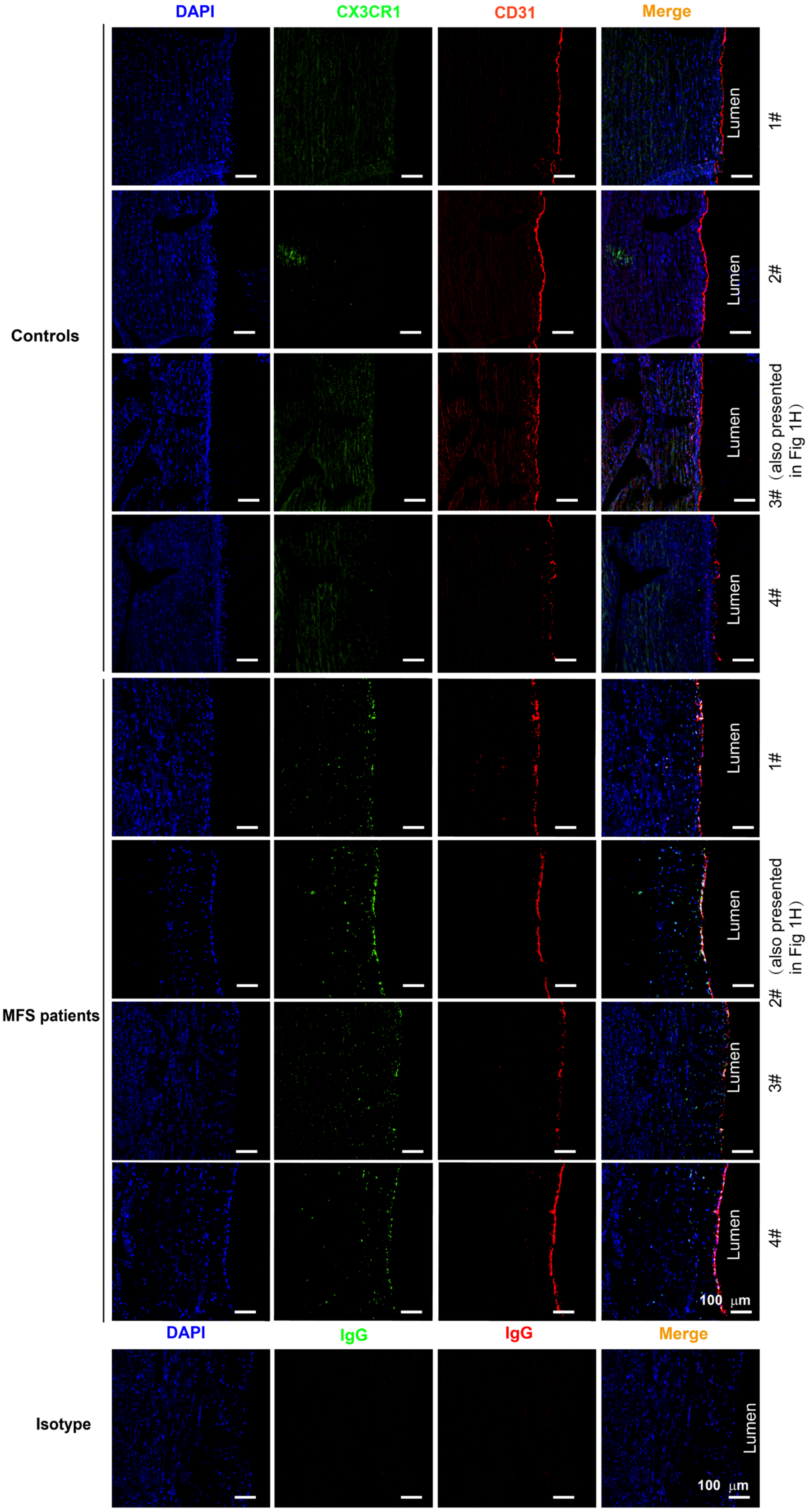

Supplementary Figure 7. Immunofluorescence staining of CX3CR1 (green) and CD31 (red) in

cross-sections of normal aortic tissues from control individuals (Controls, N=4) and aneurysmal tissues from MFS patients (N=4). The nuclei were stained blue with DAPI. Scale bar=100 μm. Mouse IgG (green) and rabbit IgG (red) as negative isotype controls.

Supplementary Figure 8

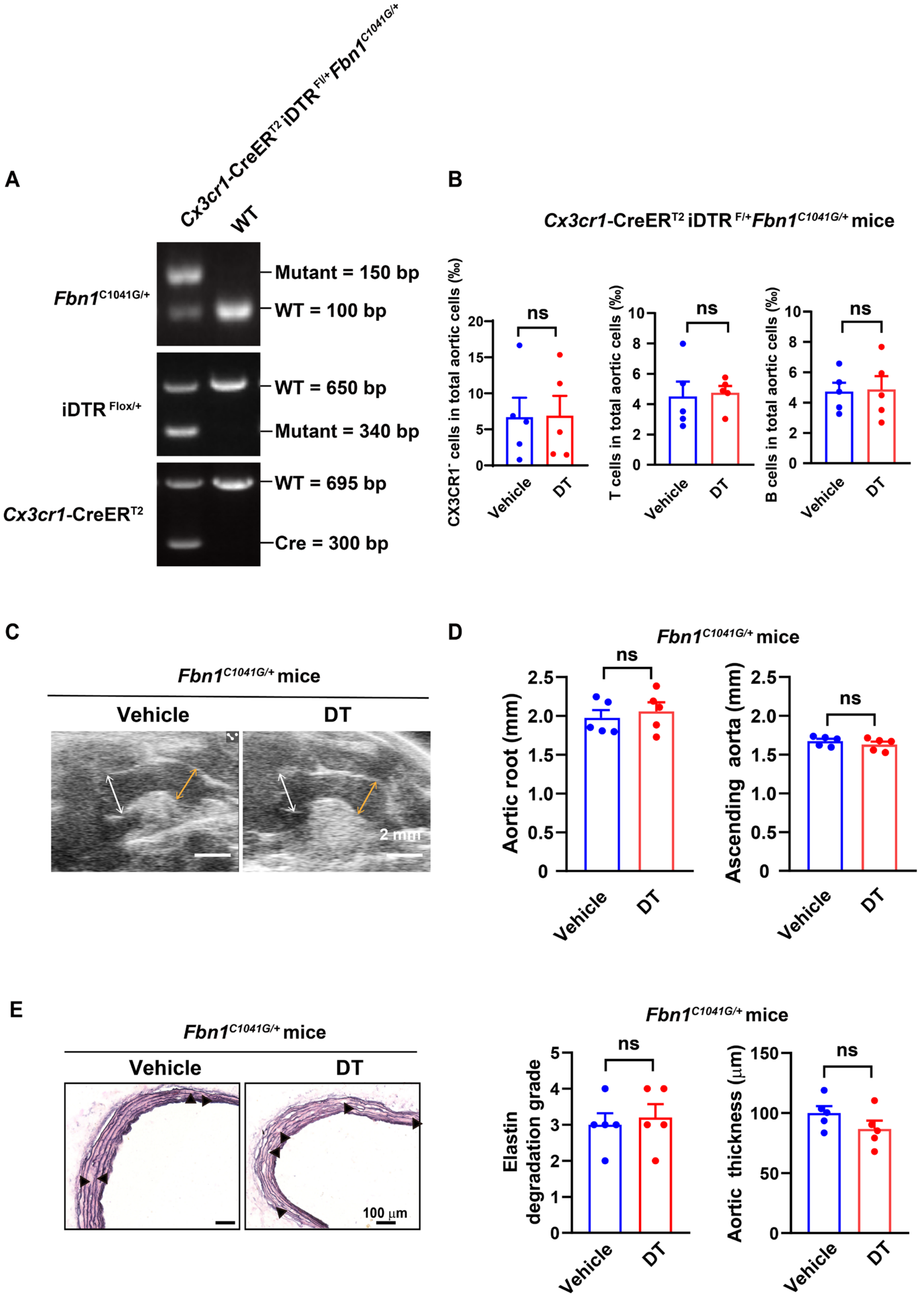

Supplementary Figure 8. A. Representative genotyping of *Cx3cr1*-CreER^{T2}iDTR$^{F/+}$*Fbn1*$^{C1041G/+}$ mice. **B.** Flow cytometry analysis of CX3CR1^{-} cells (CD45^{+}CD11b^{+}CX3CR1^{-}), T cells (CD45^{+}CD3^{+}) and B cells (CD45^{+}CD19^{+}) in aortic root and

ascending aortas from 20-week-old *Cx3cr1*-CreER^{T2}iDTR$^{F/+}$*Fbn1*$^{C1041G/+}$ mice with or without diphtheria toxin (DT)-mediated depletion. n=5, unpaired Student's *t* test, ns, no significance. **C.** Representative transthoracic echocardiographic images of aortic root and ascending aortas in 20-week-old *Fbn1*$^{C1041G/+}$ mice with or without DT treatment. Scale bar=2 mm. White arrows depict the sinus of Valsalva measurements. Yellow arrows depict ascending aorta measurement. **D.** Quantification of aortic root and ascending aorta dimeters measured by transthoracic echocardiography. n=5, unpaired Student's *t* test, ns, no significance. **E.** EVG staining of the ascending aortas in 20-week-old *Fbn1*$^{C1041G/+}$ mice with or without DT treatment. n=5, Mann-Whitney test for elastin degradation grade and unpaired Student's *t* test for aortic thickness, ns, no significance.

Supplementary Figure 9

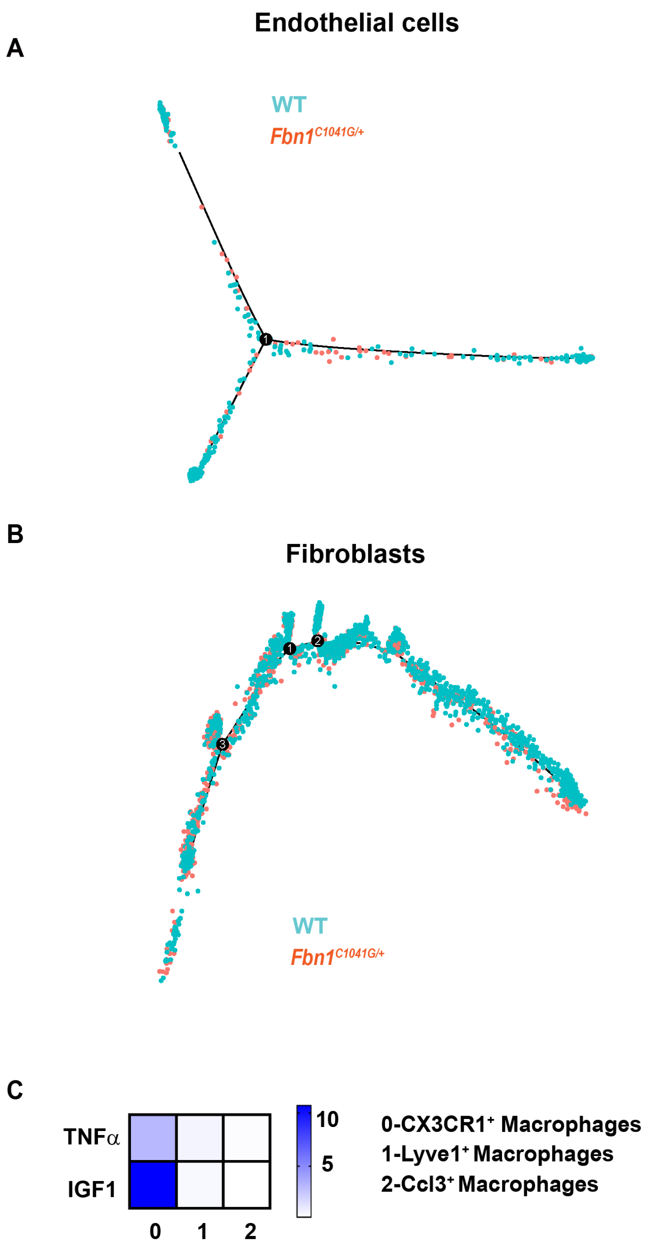

Supplementary Figure 9. A-B. The pseudotime paths of endothelial cell **(A)** and fibroblast **(B)** transcriptomes in scRNA-seq data from WT and *Fbn1*$^{C1041G/+}$ mice. **C.** scRNA-Seq data in the alterations of TNFα and IGF1 expression in three clusters macrophages based on the comparison of *Fbn1*$^{C1041G/+}$ *vs.* WT samples.

Supplementary Figure 10

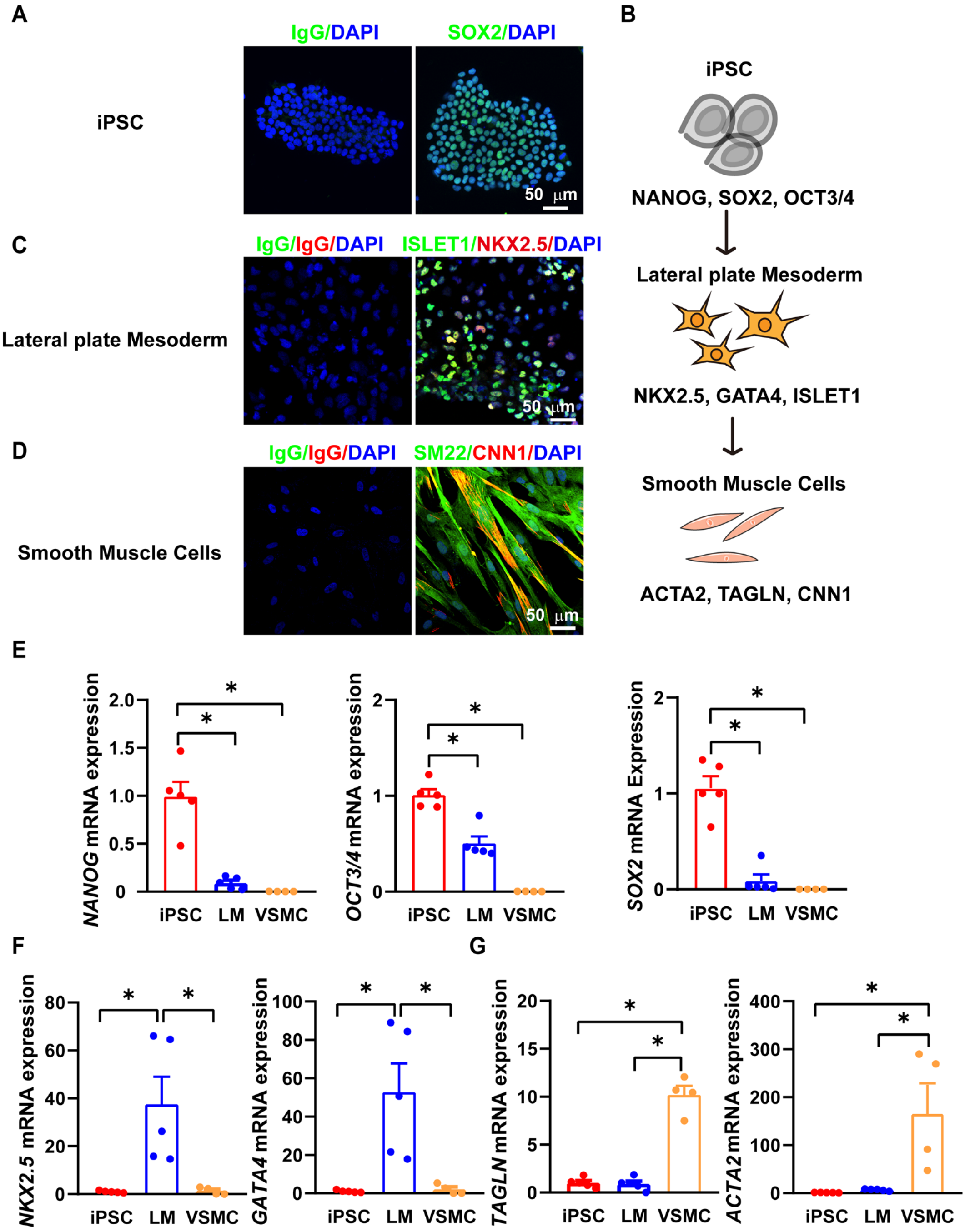

Supplementary Figure 10. Generation of MFS patient-specific iPSC-derived VSMCs. A. Immunofluorescence staining of Sox2 (green) in MFS patient-specific iPSCs. Goat IgG was applied as a negative control. The nuclei were stained blue with DAPI. Scale bar=50 μm. **B.** The experimental workflow for the generation of MFS patient-specific iPSC-derived VSMCs. **C.**

Immunofluorescence staining of ISLET1 (green) and NKX2.5 (red) in lateral plate mesoderm (LM) cells differentiated from iPSCs following FGF2 (20 ng/ml) and BMP4 (50 ng/ml) treatment for 3-4 days. Rabbit IgG (green) and mouse IgG (red) were used as negative controls. The nuclei were stained blue with DAPI. Scale bar=50 µm. **D.** Immunofluorescence staining of SM22 (green) and CNN1 (red) in VSMCs differentiated from LM cells following PDGF-BB (10 ng/ml) combined with TGF-β (2 ng/ml) treatment for 12 days. Rabbit IgG (green) and mouse IgG (red) were applied as negative controls. The nuclei were stained blue with DAPI. Scale bar=50 µm. **E-G**. Real-time PCR of gene expression in iPSCs, iPSC-differentiated LM cells and iPSC-derived VSMCs. n=5 for iPSCs and iPSC-differentiated LM cells, n=4 for iPSC-derived VSMCs. *P<0.05 by one-way ANOVA followed by Tukey's test for post hoc comparison.

Supplementary Figure 11

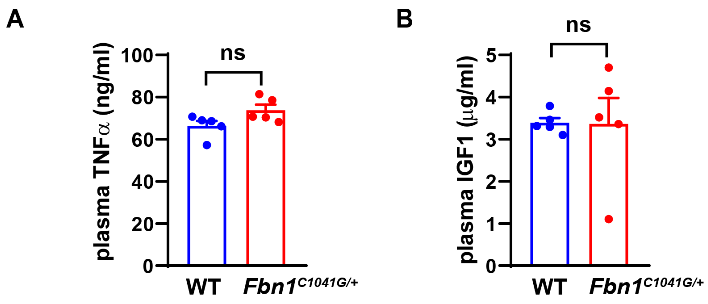

Supplementary Figure 11. ELISA measurements of plasma TNFα (A) and IGF1 (B) in 20-week-old WT and *Fbn1*$^{C1041G/+}$ mice, n=5, ns, no significance by unpaired Student's *t* test.

Supplementary Figure 12

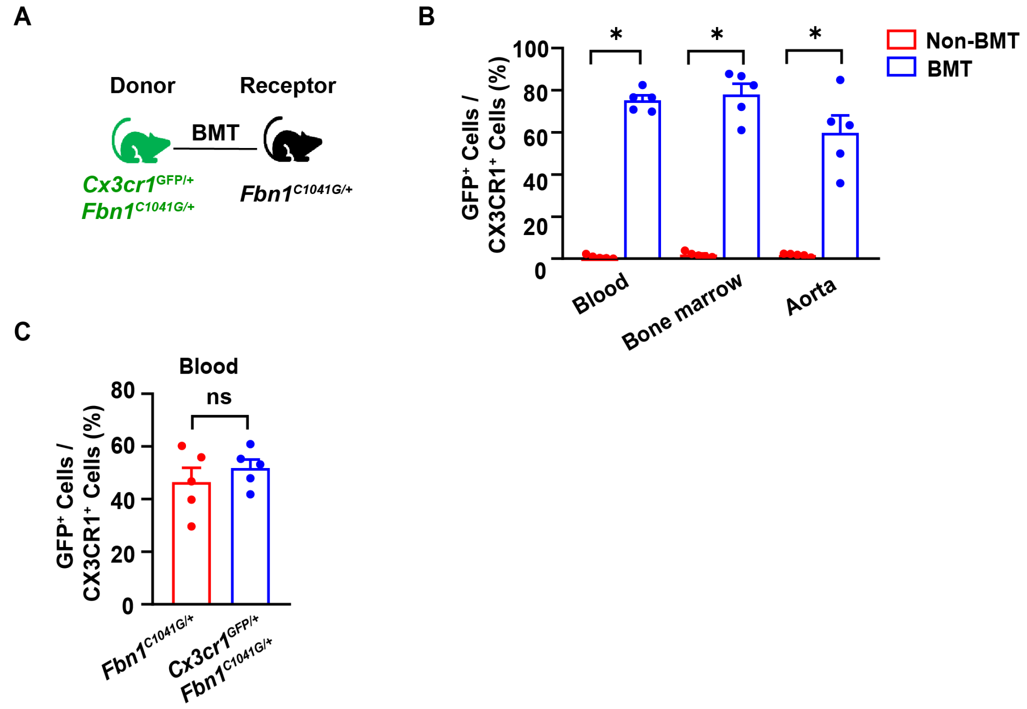

Supplementary Figure 12. A. Schematics of bone marrow transplantation from *Cx3cr1*$^{GFP/+}$*Fbn1*$^{C1041G/+}$ mice to *Fbn1*$^{C1041G/+}$ mice. **B.** Flow cytometry analysis of GFP-expressed CX3CR1^{+} cells in blood, bone marrow and aortic root and ascending aortas from *Fbn1*$^{C1041G/+}$ mice transplanted with *Cx3cr1*$^{GFP/+}$*Fbn1*$^{C1041G/+}$ bone marrow (BMT). *Fbn1*$^{C1041G/+}$ mice without bone marrow transplantation (non-BMT) were used as negative controls. n=5, *P<0.05 by unpaired Student's t test. **C.** Flow cytometry analysis of the GFP^{+} cell percentages in CX3CR1^{+} cells in the peripheral blood from *Fbn1*$^{C1041G/+}$ and *Cx3cr1*$^{GFP/+}$*Fbn1*$^{C1041G/+}$ parabionts. n=5, ns: no significance by unpaired Student's t test.

Supplementary Figure 13

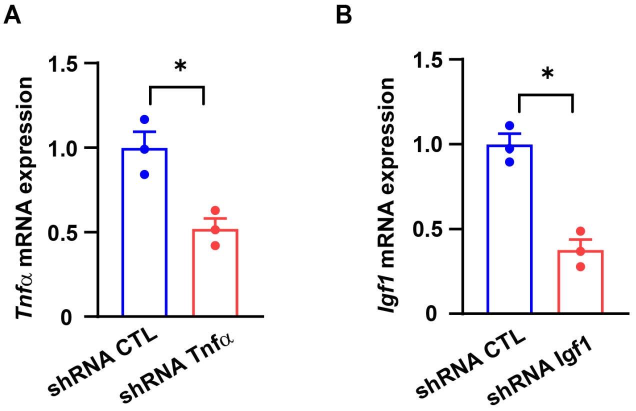

Supplementary Figure 13. Real-time PCR of Igf1 and Tnfα expression in CX3CR1^{+} macrophages isolated from 24-week-old *Fbn1*$^{C1041G/+}$ mice with transplantation of shRNA lentivirus-infected bone marrow cells. n=3, **P*<0.05 by unpaired Student's *t* test.

Supplementary Figure 14

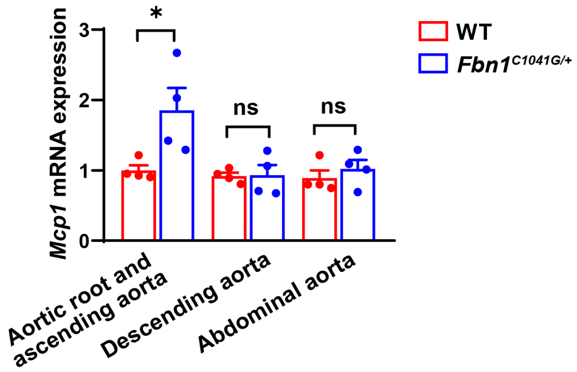

Supplementary Figure 14. Real-time PCR of Mcp1 expression in distinct segments of aortas from 3-4-week-old WT and *Fbn1*$^{C1041G/+}$ mice. n=4, **P*<0.05 by Two-way ANOVA followed by Tukey's test for post hoc comparison, ns, no significance.

Supplementary Figure 15

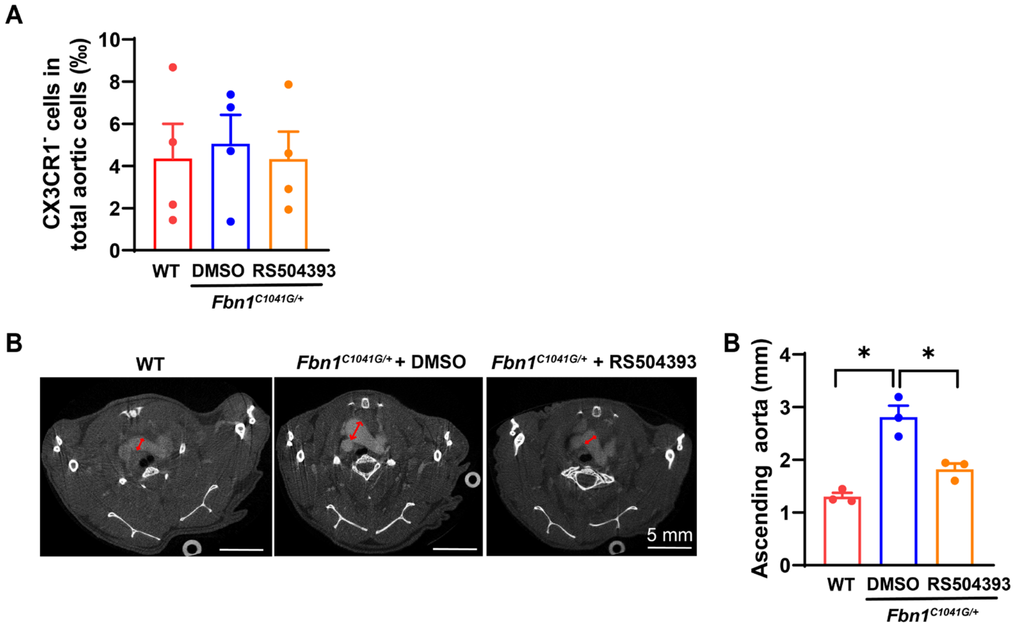

Supplementary Figure 15. A. Six-week-old *Fbn1*$^{C1041G/+}$ mice were intraperitoneally injected with the CCR2 inhibitor RS504393 (4 mg/kg/day) until aging at 26 weeks. Flow cytometry analysis of CX3CR1$^-$ cells (CD45$^+$CD11b$^+$CX3CR1$^-$) in aortic root and ascending aortas. One-way ANOVA followed by Tukey's test for post hoc comparison. n=4 for each group. **B.** Twenty-four-week-old *Fbn1*$^{C1041G/+}$ mice were intraperitoneally injected with the CCR2 inhibitor RS504393 (4 mg/kg/day) until aging at 36 weeks. Age-matched WT mice were used as a normal control. Representative cross-sectional microCT images of ascending aortas. The red double arrows indicate the maximal diameters measured in the ascending aortas. One-way ANOVA followed by Tukey's test for post hoc comparison. *P<0.05, n=3 for each group.

Supplementary Table 1: Characteristics of WT and *Fbn1*$^{C1041G/+}$ mice.

**Group**	WT	*Fbn1*$^{C1041G/+}$
**Number**	7	8
**Age (week)**	20	20
**Weight (g)**	31.71 ± 0.87	29.75 ± 0.62
**SBP (mmHg)**	104.00 ± 2.25	102.80 ± 1.83
**DBP (mmHg)**	83.57 ± 2.29	81.88 ± 0.72

Supplementary Table 2: The information of control individuals and MFS patients donating samples for aortic immunofluorescence staining.

Number	Control 1	Control 2	Control 3	Control 4	MFS 1	MFS 2	MFS 3	MFS4
**Age (year)**	54	28	13	62	15	14	33	26
**Sex**	Female	Male	Male	Male	Male	Male	Female	Male
**Diagnosis**	Dilated cardiomyopathy	Arrhythmia cardiomyopathy	Dilated cardiomyopathy	Dilated cardiomyopathy	Aortic aneurysm A3C, Aortic root aneurysm	Aortic root aneurysm,	thoracoabdominal aortic aneurysm (Crawford Type III)	Aortic root aneurysm, Aortic regurgitation
**Surgery**	Heart transplantation	Heart transplantation	Heart transplantation	Heart transplantation	Bentall procedure	David procedure	TAAA repair	Bentall procedure
**Family history**	None	None	None	None	Deny	Deny	Deny	Yes
**Diameter of aorta (at the lesion site)**	N/A	N/A	N/A	N/A	9 cm	5 cm	5.5 cm	5.6 cm
**Hypertension**	NO	NO	NO	NO	NO	NO	NO	NO
**Diabetes**	NO	NO	NO	YES	NO	NO	NO	NO
**Smoking**	NO	YES	NO	NO	NO	NO	NO	NO
**Medications**	Torsemide; Sacubactril valsartan	NO	Furosemide, Spironolactone	Glucobay, Spironolactone, Torasemide, rh-BNP, betaloc	NO	NO	NO	NO
**Height (cm)**	159	173	153	162	196	190	172	193
**Weight (kg)**	57	73	32	47	77.5	70	80	86
**Other Disease**	Atrial fibrillation	Tricuspid regurgitation	None	None	None	Lens subluxation	None	None

Supplementary Table 3: Characteristics of *Cx3cr1*-CreER^{T2}iDTR$^{F/+}$*Fbn1*$^{C1041G/+}$ mice with Vehicle or DT treatment.

**Group**	Vehicle	DT
**Number**	10	8
**Age (week)**	21	21
**Weight (g)**	31.80 ± 0.89	31.13 ± 0.69
**SBP (mmHg)**	103.10 ± 1.85	103.80 ± 2.27
**DBP (mmHg)**	85.50 ± 2.41	83.50 ± 2.67

Supplementary Table 4: Characteristics of *Fbn1*$^{C1041G/+}$ mice with PBS or DT treatment.

**Group**	PBS	DT
**Number**	5	5
**Age (week)**	20	20
**Weight (g)**	31.40 ± 1.29	30.40 ± 1.08
**SBP (mmHg)**	106.20 ± 1.36	107.20 ± 1.72
**DBP (mmHg)**	81.80 ± 2.88	83.40 ± 2.38

Supplementary Table 5: The information of MFS patients donating samples for sorting CX3CR1^{+} macrophages.

Number	MFS 1	MFS 2	MFS 3	MFS 4	MFS 5	MFS 6
**Age (year)**	34	32	24	27	35	22
**Sex**	Female	Male	Female	Male	Female	Male
**Diagnosis**	thoracoabdominal aortic aneurysm (Crawford Type III)	thoracoabdominal aortic aneurysm, Stanford type B aortic dissection	Aortic root aneurysm, Stanford type B aortic dissections	thoracoabdominal aortic aneurysm, (Crawford Type III)	thoracoabdominal aortic aneurysm (Crawford Type III)	Aortic root aneurysm
**Surgery**	TAAA repair; Common iliac artery repair	TAAA repair; Common iliac artery repair	Bentall procedure + total arch replacement	TAAA repair	TAAA repair; Common iliac artery repair	Bentall procedure
**Family history**	Deny	Yes	Yes	Yes	Yes	Deny
**Diameter of aorta (at the lesion site)**	TA: 5.5 cm, AA: 5 cm, Left iliac artery: 2 cm	Descending aorta and AA: 6 cm	Aortic sinus:6 cm, Ascending aorta:4 cm	TA: 5 cm, AA: 4 cm, Left or Right iliac artery: 1 cm	TA: 5 cm, AA: 7 cm, left/right iliac 1.5 cm/ 2 cm	Root:5.5 cm, Ascending aorta:3 cm
**Hypertension**	Yes	NO	NO	NO	Yes	NO
**Diabetes**	NO	NO	NO	NO	NO	NO
**Smoking**	NO	Yes	NO	NO	NO	NO
**Medications**	NO	NO	NO	NO	Betaloc, Norvasc	NO
**Height (cm)**	183	192	167	191	173	176
**Weight (kg)**	75	83	58	88	69	75
**Other Disease**	Spontaneous pneumothorax, Pulmonary bullae (Left)	None	None	None	None	Lens subluxation, Pulmonary bullae (left and right)

All patients were consistent with critical appraisal of the revised Ghent criteria for diagnosis of Marfan syndrome

TAAA: Thoracoabdominal aortic aneurysm; TA: thoracic aorta; AA: abdominal aorta.

Supplementary Table 6: Characteristics of *Fbn1*$^{C1041G/+}$ mice with BMT of shRNA-silenced bone marrow cells.

**Group**	shRNA CTL	shRNA Tnfα	shRNA Igf1
**Number**	5	5	5
**Age (week)**	24	24	24
**Weight (g)**	29.00 ± 1.27	29.80 ± 1.36	27.60 ± 1.03
**SBP (mmHg)**	107.20 ± 1.93	108.40 ±1.72	105.40 ± 1.50
**DBP (mmHg)**	84.80 ± 1.85	87.20 ± 1.24	82.40 ± 2.23

Supplementary Table 7: Characteristics of *Fbn1*$^{C1041G/+}$ mice with DMSO or CCR2 inhibitor RS504393 treatment. *Fbn1*$^{C1041G/+}$ mice were treated from the age of 6 weeks.

**Group**	WT mice	Fbn1$^{C1041G/+}$ mice with DMSO	Fbn1$^{C1041G/+}$ mice with RS504393
**Number**	4	8	8
**Age (week)**	26	26	26
**Weight (g)**	32.50 ± 1.32	32.13 ± 1.17	31.00 ± 0.73
**SBP (mmHg)**	107.80 ± 2.25	104.530 ±1.46	106.60 ± 1.43
**DBP (mmHg)**	82.50 ± 1.94	83.13 ± 1.58	80.50 ± 1.58

Supplementary Table 8: Characteristics of *Fbn1$^{C1041G/+}$* mice with DMSO or CCR2 inhibitor RS504393 treatment. *Fbn1$^{C1041G/+}$* mice were treated from the age of 24 weeks.

**Group**	WT mice	Fbn1$^{C1041G/+}$ mice with DMSO	Fbn1$^{C1041G/+}$ mice with RS504393
**Number**	5	5	5
**Age (week)**	36	36	36
**Weight (g)**	37.20 ± 0.97	37.00 ± 1.30	35.60 ± 1.36
**SBP (mmHg)**	107.60 ± 2.08	104.20 ±2.60	108.20 ± 2.99
**DBP (mmHg)**	81.60 ± 2.40	79.20 ± 1.86	81.40 ± 2.58

Supplementary Table 9: The primers targeting genes used for real-time PCR.

Target	Forward primer (5'-3')	Reverse primer (5'-3')
Human-18S	GCAATTATTCCCCATGAACG	GGGACTTAATCAACGCAAGC
Human-NANOG	CAGCCCCGATTCTTCCACCAGTCCC	CGGAAGATTCCCAGTCGGGTTCACC
Human-OCT3/4	GACAGGGGGAGGGGAGGAGCTAGG	CTTCCCTCCAACCAGTTGCCCCAAAC
Human-SOX2	GGGAAATGGGAGGGGTGCAAAAGAGG	GGGAAATGGGAGGGGTGCAAAAGAGG
Human-NKX2.5	TGGAGAAGACAGAGGCGGACAA	ATAGACCTGCGCCTGCGAGAA
Human-GATA4	TAAATCTAAGACGCCAGCAG	TGCCCATAGTGAGATGACAG
Human-TAGLN	TCTTTGAAGGCAAAGACATGG	TTATGCTCCTGCGCTTTCTT
Human-ACTA2	TGTGTGACAATGGCTCTGGG	CACCATCACCCCCTGATGTC
Human-IL6	ACAAGCGCCTTCGGTCCAGTT	TTCGTTCTGAAGAGGTGAGTGGCT
Human-CXCL2	CTTGTCTCAACCCCGCATCG	TTGGATTTGCCATTTTTCAGCATC
Human-MCP1	CAGATGCAATCAATGCCCCA	GACACTTGCTGCTGGTGATTC
Human-TNF	TCTTCTCGAACCCCGAGTGA	ATGAGGTACAGGCCCTCTGA
Mouse-18S	CGTCTGCCCTATCAACTTTCG	GCCTGCTGCCTTCCTTGG
Mouse-Mcp1	TTAAAAACCTGGATCGGAACCAA	GCATTAGCTTCAGATTTACGGGT
Mouse-Tnf	CCTGTAGCCCACGTCGTAG	CCTGTAGCCCACGTCGTAG
Mouse-Igf1	CTGGACCAGAGACCCTTTGC	CTGGACCAGAGACCCTTTGC
Mouse-Il6	CCAAGAGGTGAGTGCTTCCC	CCAAGAGGTGAGTGCTTCCC
Mouse-Cxcl2	CCAACCACCAGGCTACAGG	GCGTCACACTCAAGCTCTG

Supplementary Table 10: The shRNA sequences encoded in lentiviral GV493 vector.

Group	shRNA sequence
**shRNA CTL**	TTCTCCGAACGTGTCACGT
**shRNA Tnfα**	GCCGATTTGCTATCTCATACC
**shRNA Igf1**	TGATCTGAGGAGACTGGAGAT

Supplementary Methods

Measurement of Mouse Aortic Root and Ascending Aortas

For *Fbn1*$^{C1041G/+}$ MFS mice, baseline and monthly echocardiograms were performed until the time of sacrifice. All echocardiograms were performed in mice using a Visual Sonics Vevo 2200 imaging system (VisualSonics, Toronto, Canada) strictly following the well-established protocol (1-3). Mice were anesthetized using isoflurane; dose was titrated at 2–3% wt/vol isoflurane with 2 L/min O_2 to maintain heart rate 400 − 500 beats/min as monitored on concurrent 3-lead electrocardiogram. Frame rate was ≥300 frames/s, and 300 frames were stored per cine-loop. Aortic images were acquired from a modified right parasternal long axis view (1 − 2 ribs caudal to the right parasternal long axis view). The probe angled 45° relative to the chest to avoid sternum artefacts was gently moved using the X- and Y-axis stage knob to capture the longitudinal aortic image with the largest possible diameter. Images were standardized to include visualization of 2 anatomical landmarks: the innominate artery and aortic valve. The ascending aorta was defined as the region between the sinotubular junction and the innominate artery. The largest aortic luminal diameter was measured between the inner edge to inner edge of the vessel at the end diastole from three separate heart beats. Aortic images were analyzed by 2 independent observers blinded to the experimental groups.

Aortic Morphological Analysis

The hearts were dissected with the thoracic aortas from MFS mice following anesthesia using tribromoethanol (15 μL/g) and subsequent euthanasia using saturated potassium chloride (KCl) solution to arrest the heart in diastole, enabling the ex vivo evaluation of ascending aorta expansion. Aortic root and ascending aortas were further dissected and embedded in OCT compound and then

cut into frozen serial sections (7 μm thick, approximately 400 μm apart). The wall thickness and architecture were analyzed using 7 μm cross-sections stained with EVG. The wall thickness at 8 different representative locations was measured and averaged by an observer blinded to genotype and treatment for each mouse. Elastin degradation was graded as 1, <25% degradation; 2, 25% to 50% degradation; 3, 50% to 75% degradation; or 4, >75% degradation.

Blood Pressure Measurements

Mouse blood pressure (BP) was measured according to the recommendation of the American Heart Association council on high blood pressure research(4). A noninvasive computerized CODA tail-cuff blood pressure system (Kent Scientific, Torrington, CT, USA) was used to measure mouse BP. The equipment was kept clean and free from foreign scents and blood odors. The investigator was blinded to the experimental groups when performing the measurements, and the mice were tested in a randomized order. All mice underwent training sessions from 1 to 4 PM on 7 consecutive days to become accustomed to the tail-cuff procedure. After the training period, BP was measured at 1 day before sacrifice in each mouse cohort. Fifteen consecutive systolic and diastolic BP measurements were made, and the last 10 readings per mouse were recorded and averaged.

Aortic Dissociation

The mice were sacrificed by sodium pentobarbital, followed by perfusion with cold PBS. The aortic root and ascending aortas were collected in a tube with 1 mL Kreb's buffer on ice. Perivascular adipose tissue was carefully removed. After collecting the arteries, they were washed with PBS three times, and cutted into 1-2 mm^2 then digested with aortic dissociation enzyme solution (collagenase

I, 450 U/mL; hyaluronidase type I-S, 60 U/mL; DNase I, 60 U/mL; collagenase XI, 125 U/mL). Digestion was performed at 37 °C on a shaker for 1-2 h, and the tube was gently inverted every 15 mins (5). Detached cells were collected until the tissue dissolved completely. After sufficient digestion, the single-cell suspension was strained through a 70-μm strainer. The collected cells were centrifuged for 10 min at 700 g at 4 °C. For scRNA-seq, single-cell suspensions from 3 mice were pooled as one sample. For other experiments, single-cell suspensions from each mouse were evaluated individually.

Single-cell RNA Sequencing

Single-cell suspensions of aortic root and ascending aortas from WT and *Fbn1*$^{C1041G/+}$ mice were stained with a LIVE/DEAD Fixable Near-IR Dead Cell Stain Kit (1:1000) and Hoechst 33342 (Invitrogen, H3570; 1:1000) for 20 mins on ice. After being washed in PBS, the cells were resuspended in PBS. Single live cells (Hoechst^{+}/Dead Cell Stain^{-}) were sorted into PBS with 0.04% BSA using a BD FACS Symphony S6 (BD Biosciences). A Chromium Single Cell 3' Reagent Kit v3 was used, and Standard 10X Chromium Single Cell 3' v3 (10X Genomics GemCode Technology) protocols were followed for scRNA-seq. Briefly, single cells with specific 10X barcodes and unique molecular identifiers were generated by partitioning the cells into Gel Bead-in-Emulsions. A cDNA library was generated and sequenced with a NovaSeq (S4) 6000 instrument (PE150). Raw sequencing data were processed using CellRanger (version 3.1) to demultiplex raw data and generate FASTQ files, and the resulting data were aligned and counted with CellRanger pipelines.

scRNA-seq Data Analysis

General analysis and visualization

The R package Seurat (version 3.2.2) was used for analyzing scRNA-seq data. Briefly, gene features expressed in at least three cells and cells with at least 100 detected genes were retained; then, cells displaying fewer than 500 or more than 4000 gene features or having more than 20% mitochondrial counts were filtered (Supplementary Figure 2). After passing the quality control metrics, 6,508 cells in the WT group and 3,827 cells in the *Fbn1*$^{C1041G/+}$ group were further analyzed. Then, gene expression was normalized by "LogNormalize". Integrative analysis of the two groups was performed using canonical correlation analysis to remove batch effects. A standard integrated analysis was performed with default parameters. The top 2000 highly variable genes were selected and scaled by "ScaleData". Principal component analysis was then performed, and the first 15 principal components with a resolution of 0.2 were used for unbiased clustering. Clusters were visualized with t-SNE or UMAP. The markers for every cluster compared to all remaining cells were identified by "FindAllMarkers" (min.pct = 0.25, logfc.threshold = 0.25, only.pos = TRUE) and were used in the following analyses. Reported Padjusted values were calculated using Benjamini–Hochberg correction. Heatmaps of scRNA-seq data were plotted with the R package ggplot2.

Focused analysis of specific cell clusters

Leukocytes were extracted by "subsets." using marker genes Igkc, Myl7 and Itgb2, and unbiased clustering was performed again with a resolution of 1.5. To improve the accuracy of naming the subclusters, clusters with an average of fewer than 15 cells per group were removed.

Analysis of cell trajectory

Monocle2 (v2.6.0) was used to study the pseudotime trajectories of cells. The UMI matrix was used as input, and variable genes detected by Seurat were used for building traces. Branches in the cell trajectory represent cells that have alternative gene expression patterns.

Ligand–receptor interaction

The analysis of ligand–receptor interactions between intimal CX3CR1$^+$ macrophages and VSMC clusters was performed based on the CellPhoneDB database (6). Membrane receptors and ligands were selected according to cluster-specific genes within VSMCs and intimal CX3CR1$^+$ macrophage clusters, respectively. All potential pairs of ligand–receptor interactions were identified according to a prebuilt protein–protein interaction library. The expression of interaction pairs between two clusters was calculated as the product of the mean expression of the ligand in the ligand-producing cluster and the mean expression of the receptor in the receptor-producing cluster.

DEGs identification

After the gene expression were normalized by "NormalizeData." from the Seurat package (Seurat v3.0), selection. "FindVariableFeatures" was used to find feature variables. Each gene's expression level was scaled by "ScaleData" and set "vars.to.regress=percent.mito" for considering the total number of unique molecular identifiers (UMI) and percentage of mitochondria content within each cell. Finally, we used "FindMarkers" to identify DEGs between *Fbn1*$^{C1041G/+}$ and WT VSMCs. The thresholds for the final DEG were set as FDR-corrected P-value <0.05 and logfc.threshold=0.25.

Gene Ontology and KEGG analysis

Gene ontology and KEGG analysis was conducted using "clusterProfiler" as previous report (7).

Bone marrow transplantation

Bone marrow transplantation was performed as described in previous reports with minor modifications (8, 9). Twelve-week-old male *Fbn1*$^{C1041G/+}$ Mice were exposed to 5 Gy of X-ray radiation twice at a 4-hour interval followed by the injection of bone marrow cells from *Fbn1*$^{C1041G/+}$*Cx3cr1*$^{GFP/+}$ mice (5×10^6 cells/mouse) or shRNA lentivirus-infected bone marrow cells via the tail vein (shRNA sequences were listed in Supplementary Table 10). Lentiviral shRNA silencing of murine bone marrow cell was performed following previous studies (10, 11). Briefly, bone marrow cells were isolated from 6-week-old *Fbn1*$^{C1041G/+}$ mice and cultured using RPMI 1640 medium with 10% FBS overnight in vitro. Then, bone marrow cells were infected with lentivirus encoding control, TNFα or IGF1 shRNA at the multiplicity of infection (MOI) of 20. The infected bone marrow cells were collected for transplantation into recipient *Fbn1*$^{C1041G/+}$ mice at 12 hours post lentivirus adding. To prevent infection post bone marrow transplantation, mice were administered drinking water containing neomycin (100 mg/L) and polymyxin B sulfate (10 mg/L) for 2 weeks post radiation exposure. Mice that did not receive bone marrow injections were used as controls to confirm the lethal exposure dose, and they generally died within 2 weeks. At 12 weeks posttransplantation, blood cells and bone marrow cells from chimeric *Fbn1*$^{C1041G/+}$ mice were collected to evaluate the efficiency of bone marrow reconstitution, as demonstrated by the percentages of GFP-expressing cells in total CX3CR1^{+} cells in blood and bone marrow.

Parabiosis

Age-matched *Fbn1*$^{C1041G/+}$*Cx3cr1*$^{GFP/+}$ and *Fbn1*$^{C1041G/+}$ male mice at 6-8 weeks old were parabiosed using a method described previously (12). The parabionts were anesthetized by isoflurane (1-2%). The corresponding lateral aspects of each mouse were shaved and sterilized with 70% ethanol. A longitudinal skin incision was made from 0.5 cm above the elbow all the way to 0.5 cm below the knee joint of each mouse, and the subcutaneous fascia was bluntly dissected to create approximately 0.5 cm of free skin. The elbow and knee joints were attached by 3-0 silk suture and tied, and the dorsal and ventral skin were stitched through continuous 5-0 absorbable suture. Then, 0.5 mL of 0.9% NaCl was subcutaneously administered to each mouse to prevent dehydration. Prophylactically, mice were treated with a sulfamethoxazole (2 mg/mL)/trimethoprim (0.4 mg/mL) oral suspension in their water bottle for 10 days to prevent bacterial infections. Established shared blood circulation was confirmed by flow cytometry analysis of GFP-expressed CX3CR1$^+$ cells in the blood from parabiotic mice at 4 weeks after surgery. After blood cross-circulation between parabionts was confirmed, the parabionts were further bred for 8 weeks and then euthanized to compare the numbers of GFP-expressing CX3CR1$^+$ macrophages in the aortic root and ascending aortas between these parabionts by immunofluorescence staining and flow cytometry.

In vivo labeling of intimal CX3CR1$^+$ macrophages

Twelve-week-old *Fbn1*$^{C1041G/+}$ mice were injected with 5 μg/ mice rat-anti-mouse CD45 (30-F11) antibody (SY65087-1; Cloud-clone corp, Wuhan) or mouse IgG antibody diluted in sterile PBS intravenously to pre-label the lumen-facing side cells for 30 minutes prior to sacrifice. Then, aortic

root and ascending aorta was dissected and digested into single-cell suspension, followed by the incubation of PE-conjugated anti-mouse CX3CR1 antibody and Alexa Fluor 488-conjugated anti-rat IgG secondary antibody. Flow cytometry analysis were performed to detect CX3CR1^{+} macrophages labelled with anti-CD45 antibody.

MFS-iPSC derivation and VSMC differentiation

Peripheral blood mononuclear cells (PBMCs) from MFS patients were reprogrammed using a CytoTuneTM-iPS 2.0 A Sendai Reprogramming Kit (Thermo Fisher Scientific) according to the manufacturer's instructions. iPSCs were maintained on Matrigel (Corning) with mTeSR (STEMCELL) medium. Cells were routinely passaged with EDTA (0.04%). Since second heart field-derived VSMCs exhibit more significant aneurysm-related pathological alterations compared with neural crest-derived cells(13-15), the protocol of lateral plate mesoderm (equivalent to second heart field)-derived VSMC differentiation from MFS patient-specific iPSCs was selected and performed according to Cheung's protocol with a modification (16-18). Briefly, iPSCs were passaged into 100-150 cell cluster sizes, and then the mTeSR medium was changed to basal medium containing FGF2 (20 ng/mL), LY294002 (10 μM), and BMP4 (10 ng/mL) for 2 days. Cells were then cultured in FGF2 (20 ng/mL) and BMP4 (50 ng/mL) for another 3-4 days to generate lateral mesoderm cells. The medium was changed with PDGF-BB (10 ng/mL) combined with TGF-β (2 ng/mL) at an intermediate cell density of 60%. Cells were differentiated into VSMCs for 12 days, and half of the medium was changed every other day. The MFS-specific iPSC-derived VSMCs were cultured in DMEM with 10% fetal bovine serum, and passages 4-7 were used in the experiments.

Contrast-enhanced micro-computed tomography imaging

Mouse ascending aortas were *in vivo* scanned using contrast-enhanced micro-computed tomography (microCT, SkyScan 1276, Bruker). The contrast agent iohexol solution (0.7 mg/ml) was injected through the tail vein at the speed of 166 μl/min for 5 minutes using microfluidic pumps. The scanner was set at a voltage of 60 kV, a current of 200 μA and a resolution of 13 μm per pixel and the results were analyzed according to the manufacturer's instructions. The image reconstruction software (NRecon v1.6), data analysis software (CTAn v1.9) and three-dimensional model visualization software (mCT Vol v2.0) were applied to analyze the diameter of ascending aorta.

Supplementary References

1. Chen JZ, Sawada H, Moorleghen JJ, Weiland M, Daugherty A, and Sheppard MB. Aortic Strain Correlates with Elastin Fragmentation in Fibrillin-1 Hypomorphic Mice. *Circ Rep.* 2019;1(5):199-205.
2. Sawada H, Chen JZ, Wright BC, Moorleghen JJ, Lu HS, and Daugherty A. Ultrasound Imaging of the Thoracic and Abdominal Aorta in Mice to Determine Aneurysm Dimensions. *J Vis Exp.* 2019(145).
3. Sawada H, Katsumata Y, Higashi H, Zhang C, Li Y, Morgan S, et al. Second Heart Field-Derived Cells Contribute to Angiotensin II-Mediated Ascending Aortopathies. *Circulation.* 2022;145(13).
4. Kurtz TW, Griffin KA, Bidani AK, Davisson RL, and Hall JE. Recommendations for Blood Pressure Measurement in Humans and Experimental Animals. *Arteriosclerosis, Thrombosis, and Vascular Biology.* 2005;25(3).
5. Galkina E, Kadl A, Sanders J, Varughese D, Sarembock IJ, and Ley K. Lymphocyte recruitment into the aortic wall before and during development of atherosclerosis is partially L-selectin dependent. *J Exp Med.* 2006;203(5):1273-82.
6. Cai J, Deng J, Gu W, Ni Z, Liu Y, Kamra Y, et al. Impact of Local Alloimmunity and Recipient Cells in Transplant Arteriosclerosis. *Circ Res.* 2020;127(8):974-93.
7. Yu G, Wang L-G, Han Y, and He Q-Y. clusterProfiler: an R Package for Comparing Biological Themes Among Gene Clusters. *OMICS: A Journal of Integrative Biology.* 2012;16(5):284-7.
8. Seimon TA, Wang Y, Han S, Senokuchi T, Schrijvers DM, Kuriakose G, et al. Macrophage deficiency of p38alpha MAPK promotes apoptosis and plaque necrosis in advanced atherosclerotic lesions in mice. *J Clin Invest.* 2009;119(4):886-98.
9. Duran-Struuck R, and Dysko RC. Principles of bone marrow transplantation (BMT): providing optimal veterinary and husbandry care to irradiated mice in BMT studies. *J Am Assoc Lab Anim Sci.* 2009;48(1):11-22.
10. Bot I, Guo J, Van Eck M, Van Santbrink PJ, Groot PHE, Hildebrand RB, et al. Lentiviral shRNA silencing of murine bone marrow cell CCR2 leads to persistent knockdown of CCR2 function in vivo. *Blood.* 2005;106(4):1147-53.
11. Qu J, and Yang Z. Protocol to produce high-titer retrovirus for transduction of mouse bone marrow cells. *STAR Protoc.* 2021;2(2).
12. Ardehali R, Weissman IL, Ali SR, Zhao P, Sereti K-I, and Kamran P. Parabiosis in Mice: A Detailed Protocol. *J Vis Exp.* 2013(80):e50556 1-5.
13. Sawada H, Katsumata Y, Higashi H, Zhang C, Li Y, Morgan S, et al. Second Heart Field–Derived Cells Contribute to Angiotensin II–Mediated Ascending Aortopathies. *Circulation.* 2022;145(13):987-1001.
14. Nakamura K, Dalal AR, Yokoyama N, Pedroza AJ, Kusadokoro S, Mitchel O, et al. Lineage-Specific Induced Pluripotent Stem Cell–Derived Smooth Muscle Cell Modeling Predicts Integrin Alpha-V Antagonism Reduces Aortic Root Aneurysm Formation in Marfan Syndrome Mice. *Arteriosclerosis, Thrombosis, and Vascular Biology.* 2023;43(7):1134-53.
15. Pedroza AJ, Dalal AR, Shad R, Yokoyama N, Nakamura K, Cheng P, et al. Embryologic Origin Influences Smooth Muscle Cell Phenotypic Modulation Signatures in Murine Marfan Syndrome Aortic Aneurysm. *Arteriosclerosis, Thrombosis, and Vascular Biology.* 2022;42(9):1154-68.
16. Granata A, Serrano F, Bernard WG, McNamara M, Low L, Sastry P, et al. An iPSC-derived

vascular model of Marfan syndrome identifies key mediators of smooth muscle cell death. *Nat Genet.* 2017;49(1):97-109.

17. Hotta A, Cheung AYL, Farra N, Garcha K, Chang WY, Pasceri P, et al. EOS lentiviral vector selection system for human induced pluripotent stem cells. *Nat Protoc.* 2009;4(12):1828-44.

18. Cheung C, Bernardo AS, Pedersen RA, and Sinha S. Directed differentiation of embryonic origin–specific vascular smooth muscle subtypes from human pluripotent stem cells. *Nat Protoc.* 2014;9(4):929-38.
